# The E3 ubiquitin ligase SlATL2 suppresses tomato immunity by promoting SlCSN5a degradation during *Pseudomonas syringae* pv. *tomato* DC3000 infection

**DOI:** 10.1093/hr/uhaf078

**Published:** 2025-03-10

**Authors:** Yujie Dai, Xiaodan Li, Yeling He, Liya Zhu, Yan Bi, Fengming Song, Dayong Li

**Affiliations:** Ministry of Agriculture and Rural Affairs Key Laboratory of Molecular Biology of Crop Pathogens and Insect Pests, Institute of Biotechnology, Zhejiang University, Hangzhou 310058, China; Zhejiang Key Laboratory of Biology and Ecological Regulation of Crop Pathogens and Insects, Institute of Biotechnology, Zhejiang University, Hangzhou 310058, China; State Key Laboratory for Rice Biology and Breeding, Institute of Biotechnology, Zhejiang University, Hangzhou 310058, China; Ministry of Agriculture and Rural Affairs Key Laboratory of Molecular Biology of Crop Pathogens and Insect Pests, Institute of Biotechnology, Zhejiang University, Hangzhou 310058, China; Zhejiang Key Laboratory of Biology and Ecological Regulation of Crop Pathogens and Insects, Institute of Biotechnology, Zhejiang University, Hangzhou 310058, China; State Key Laboratory for Rice Biology and Breeding, Institute of Biotechnology, Zhejiang University, Hangzhou 310058, China; Ministry of Agriculture and Rural Affairs Key Laboratory of Molecular Biology of Crop Pathogens and Insect Pests, Institute of Biotechnology, Zhejiang University, Hangzhou 310058, China; Zhejiang Key Laboratory of Biology and Ecological Regulation of Crop Pathogens and Insects, Institute of Biotechnology, Zhejiang University, Hangzhou 310058, China; State Key Laboratory for Rice Biology and Breeding, Institute of Biotechnology, Zhejiang University, Hangzhou 310058, China; Ministry of Agriculture and Rural Affairs Key Laboratory of Molecular Biology of Crop Pathogens and Insect Pests, Institute of Biotechnology, Zhejiang University, Hangzhou 310058, China; Zhejiang Key Laboratory of Biology and Ecological Regulation of Crop Pathogens and Insects, Institute of Biotechnology, Zhejiang University, Hangzhou 310058, China; State Key Laboratory for Rice Biology and Breeding, Institute of Biotechnology, Zhejiang University, Hangzhou 310058, China; Ministry of Agriculture and Rural Affairs Key Laboratory of Molecular Biology of Crop Pathogens and Insect Pests, Institute of Biotechnology, Zhejiang University, Hangzhou 310058, China; Zhejiang Key Laboratory of Biology and Ecological Regulation of Crop Pathogens and Insects, Institute of Biotechnology, Zhejiang University, Hangzhou 310058, China; State Key Laboratory for Rice Biology and Breeding, Institute of Biotechnology, Zhejiang University, Hangzhou 310058, China; Ministry of Agriculture and Rural Affairs Key Laboratory of Molecular Biology of Crop Pathogens and Insect Pests, Institute of Biotechnology, Zhejiang University, Hangzhou 310058, China; Zhejiang Key Laboratory of Biology and Ecological Regulation of Crop Pathogens and Insects, Institute of Biotechnology, Zhejiang University, Hangzhou 310058, China; State Key Laboratory for Rice Biology and Breeding, Institute of Biotechnology, Zhejiang University, Hangzhou 310058, China; Ministry of Agriculture and Rural Affairs Key Laboratory of Molecular Biology of Crop Pathogens and Insect Pests, Institute of Biotechnology, Zhejiang University, Hangzhou 310058, China; Zhejiang Key Laboratory of Biology and Ecological Regulation of Crop Pathogens and Insects, Institute of Biotechnology, Zhejiang University, Hangzhou 310058, China; State Key Laboratory for Rice Biology and Breeding, Institute of Biotechnology, Zhejiang University, Hangzhou 310058, China

## Abstract

Plant immunity involves complex regulatory mechanisms that mediate the activation of defense responses against pathogens. Protein degradation via ubiquitination plays a crucial role in modulating these defenses, with E3 ubiquitin ligases functioning as central regulators. This study investigates the role of SlATL2, an ARABIDOPSIS TÓXICOS EN LEVADURA (ATL)-type E3 ubiquitin ligase localized in the plasma membrane, in the immune response of tomato plants against *Pseudomonas syringae* pv. *tomato* (*Pst*) DC3000. Our findings demonstrate that SlATL2 expression is induced upon *Pst* DC3000 infection and treatment with defense hormones salicylic acid and jasmonic acid. Functionally, SlATL2 negatively regulates immune responses, impairing resistance to *Pst* DC3000 and suppressing flg22-triggered immunity. In addition, SlATL2 limits pathogen-induced reactive oxygen species and callose accumulation by targeting the COP9 signalosome subunit 5a (SlCSN5a), a key positive regulator of tomato defense responses against *Pst* DC3000. This interaction, which occurs via the N-terminal residue of SlATL2, results in the ubiquitination and 26S proteasomal degradation of SlCSN5a, thereby suppressing SA-dependent expression of defense response genes associated and limiting reactive oxygen species production. This work sheds light on the molecular mechanism through which the E3 ubiquitin ligase SlATL2 attenuates tomato immune responses by targeting a COP9 signalosome subunit for degradation. These discoveries deepen our insights into the post-translational mechanisms governing plant immune responses and provide fresh opportunities to bolster crop resistance against bacterial pathogens.

## Introduction

Plants defend against pathogens through a multilayered immune system [1, 2]. The first layer, pattern-triggered immunity (PTI), relies on pattern recognition receptors (PRRs) that detect pathogen-associated molecular patterns (PAMPs) and activate receptor-like cytoplasmic kinases (RLCKs) [3, 4]. This triggers mitogen-activated protein kinases (MAPKs), reactive oxygen species (ROS) bursts, calcium influx, and pathogenesis-related gene expression [1, 5, 6]. A second layer, effector-triggered immunity (ETI), occurs when nucleotide-binding site (NBS) and leucine-rich repeat (LRR) receptors (NLRs) recognize pathogen effectors [7–9]. ETI activation also depends on PRR-mediated responses like ROS production and MAPK signaling [10–12].

Ubiquitination is a widely conserved post-translational modification in eukaryotes that enables rapid and precise regulation of protein stability, directly controlling key biological processes [13]. This process involves a cascade of enzymatic activities with three main components: E1, also known as ubiquitin-activating enzymes (UBAs); the E2 ubiquitin-conjugating enzymes (UBCs); and the E3 ubiquitin ligases. The *Arabidopsis* genome encodes 2 E1 enzymes, 47 E2 enzymes, and over 1400 E3 ligases, highlighting the critical role of E3 ligases in determining substrate specificity [14]. The ARABIDOPSIS TÓXICOS EN LEVADURA (ATLs) gene family comprises a group of plant-specific E3 ligases belonging to the REALLY INTERESTING NEW GENE (RING)-type. These proteins are defined by a RING-H2-type zinc finger domain and feature one or two hydrophobic transmembrane segments at the N-terminal region [15].

Several ATLs have been recognized as key regulators in plant responses to pathogen attacks. For example, *Arabidopsis* AtATL2 is rapidly induced during infection with the necrotrophic fungus *Alternaria brassicicola* and is essential for the defense response against this pathogen [16, 17]. AtATL9 is involved in defense responses mediated by chitin and NADPH oxidase against the biotrophic pathogen *Golovinomyces cichoracearum* [18]. Similarly, *AtATL12* has been shown to mediate crosstalk between hormone signaling chitin- and NADPH oxidase-dependent immune responses [19]. Other isoforms, such as *ATL31* and *ATL6,* serve as positive regulators against bacterial pathogens by promoting the stability of BOTRYTIS-INDUCED KINASE1 (BIK1) [20, 21]. ATL31 and ATL6 enhance defense responses by facilitating the proteasomal degradation of the calcium-dependent protein kinase CPK28, which controls the turnover of BIK1 [22]. In rice, the ER-localized EL5/*OsATL24* is also involved in disease resistance [23]. In *Vitis vinifera*, 12 ATL genes have been associated with pathogen responses, particularly against biotrophic pathogens causing powdery and downy mildew, grey mold, and noble rot [24]. Additionally, *PbrATL18* in *Pyrus breschneideri* regulates resistance to *Colletotrichum fructicola* by promoting the expression of defensive enzymes, including chitinase, phenylalanine ammonia-lyase, superoxide dismutase, polyphenol oxidase, catalase, and peroxidase [25]. In Solanaceous plants, StRFP1 and NbATL60 mediate basal immunity via the PTI pathway in *Solanum tuberosum* and *N. benthamiana*, respectively [26], while *LeATL6* in *Lycopersicon esculentum* plays a prominent role in regulating defense responses by the JA signaling pathway [27, 28].

The COP9 signalosome (CSN) is an evolutionarily conserved multiprotein complex found in eukaryotes, generally consisting of eight distinct subunits (CSN1 to CSN8) across most organisms [29]. In plants, the CSN is essential for regulating Cullin-RING ubiquitin E3 ligases (CRLs), the most extensive class of ubiquitin E3 ligases [30–32]. Specifically, CSN influences CUL1-containing ligases, known as SCF complexes, that are involved in multiple signaling pathways. These SCF complexes include SCF^TIR1^, which plays a role in auxin signaling [33, 34]; SCF^UFO^, involved in flower development [35]; and SCF^COI1^ and SCF^SLY1^, which regulate jasmonate and gibberellin signaling, respectively [36–38], and SCF^CFK1^, which is critical for seedling development [39]. CSN also associated with CUL3/4 ligases in *Arabidopsis* [40, 41]. The subunit CSN5 has been shown to participate in a broad spectrum of biological processes [30, 42, 43]. In *Arabidopsis*, two homologous genes, *CSN5A* and *CSN5B*, encode CSN5 [44], and both versions are incorporated into the CSN complex, contributing to its isopeptidase function [45, 46]. *csn5a-1* and *csn5b-1* are viable but show distinct phenotypes [41, 45, 46]. While *csn5b-1* plants seem similar to wild-type (WT) under normal conditions, *csn5a-1* mutants exhibit significant growth defects, such as smaller, purple (fusca-like) seedlings, and stunted adult plants that produce fewer seeds. In addition to its developmental roles, CSN5 is implicated in plant immunity. It has been shown that *Arabidopsis* CSN5A associates with approximately 30 effector proteins from *Hyaloperonospora arabidopsidis* (*Hpa*) and *Pseudomonas syringae* (*Psy*), and *csn5a* mutants exhibit enhanced resistance to both pathogens [47], implying that *CSN5A* can modulate immune responses through effector interactions. Furthermore, the PASSE-MURAILLE (MIPM) protein from the pathogenic nematode *Meloidogyne incognita* interacts with CSN5, compromising plant immunity and weakening host resistance [48]. In *Arabidopsis*, depletion of *CSN5A* and *CSN5B* increases the activity of CUL1/3/4, ligases, suggesting that CSN5 regulates CRL activity and may impact the accumulation of R proteins [41]. Silencing *TaCSN5* in wheat boosts leaf rust resistance and increases *Pathogenesis-related 1* (*PR-1*) gene expression, with *TaCSN5* acting as a negative regulator of plant immunity [49]. In tomato plants, the CSN5 subunit physically interacts with CORONATINE INSENSITIVE1 (COI1), thereby influencing JA-dependent defense responses against necrotrophic pathogens. Interestingly, a decrease in wound response corresponds to reduced JA biosynthesis, while salicylic acid (SA) levels remain unchanged [50]. Thus, CSN5 coordinates both plant development and defense responses, particularly those linked to the JA pathway.

This research investigates the functional role and regulatory network of SlATL2, a plasma membrane-localized ATL-type E3 ligase. Our results indicate that SlATL2 suppresses tomato immune responses by facilitating the polyubiquitination and subsequent degradation of the CSN subunit CSN5a, a critical component of the ubiquitin-proteasome system (UPS). These findings unveil SlATL2 as a key negative regulator of tomato immunity, revealing the molecular mechanisms by which this E3 ubiquitin ligase modulates defense responses during *Pst* DC3000 infection.

## Results

### Identification and characterization of SlATL2 in tomato

SlATL2 (Solyc01g066430) was first identified as a gene induced by biotic stress in a tomato microarray dataset (http://ted.bti.cornell.edu/cgi-bin/TFGD/digital/home.cgi). It has also been shown to respond to abiotic stresses like salt, heat, and drought [51]. Studying SlATL2 is important for understanding how tomato plants cope with various stresses and could help improve plant resilience to environmental challenges. SlATL2, comprising 300 amino acids (aa), contains a predicted transmembrane domain (TM) at its N-terminal region, with a GLD tripeptide and a RING motif located in its central region (Supplementary Data Fig. S1A), classifying it as an ATL RING-type E3 ubiquitin ligase.

**Figure 1:**
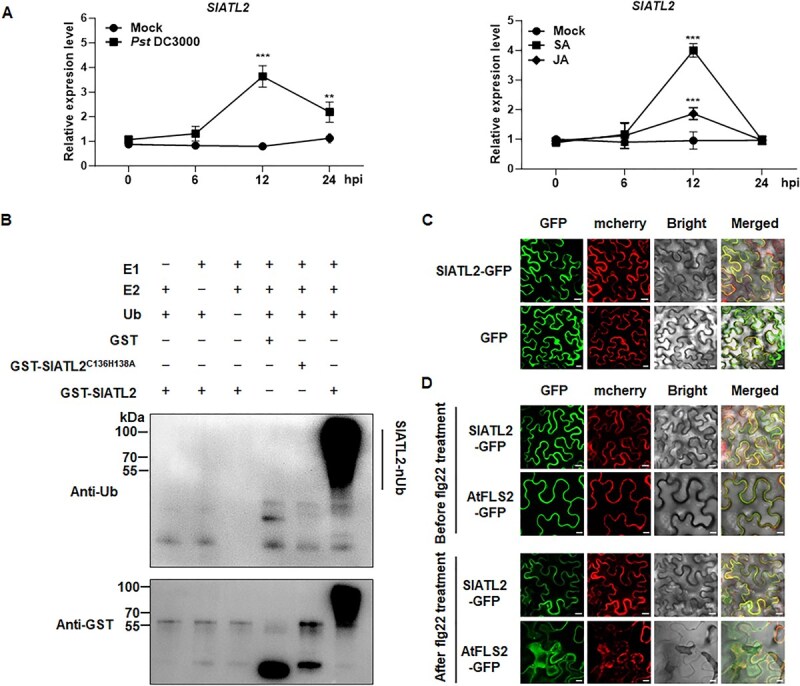
Characterization of SlATL2 through expression profiling and biochemical analysis. (A) Expression profile of *SlATL2* upon pathogen infection and defense hormones. *SlATL2* expression was quantified by qPCR, with *SlActin* employed as the reference gene. Data points represent means ± SD; n = 3. Asterisks denote statistical significance (^**^P < 0.01, ^***^P < 0.001, Student’s *t*-test). (B) The E3 ubiquitin ligase activity of SlATL2 and SlATL2^C136H138A^  *in vitro*. Recombinant glutathione S-transferase (GST)-tagged SlATL2 and SlATL2^C136H138A^ were incubated with E1, E2, and ubiquitin-FLAG. The vertical solid line indicates the ubiquitinated proteins. (C) Subcellular localization of SlATL2. mCherry represents a membrane marker fusion protein with AtPIP2A. The overlap of the GFP and mCherry fluorescence in merged images indicates the co-localization of both proteins. Bars = 20 μm. (D) Subcellular localization of SlATL2 in response to flg22 treatment. *N. benthamiana* leaves co-expressing SlATL2 and AtPIP2A were treated with flg22 (100 nM), with AtFLS2 serving as a positive control for flg22 treatment. Bars = 20 μm.

To investigate the possible function of SlATL2 in plant defense mechanisms, we initially examined its expression patterns in plants infected with *Pst* DC3000, as well as in response to treatments with SA and JA, two essential hormones that regulate defense responses in plants. In plants infected with *Pst* DC3000, *SlATL2* expression was significantly upregulated, reaching a peak at 24 h postinoculation (hpi) with a six-fold increase compared with mock-inoculated plants (Fig. 1A). Additionally, *SlATL2* expression was upregulated by SA or JA. In SA-treated plants, *SlATL2* expression showed a significant 4.1-fold increase at 12 h post-treatment compared to mock control plants. (Fig. 1A). In contrast, JA-treated plants showed a reduced but significant increase, with SlATL2 expression by 1.9-fold at 12 h compared with the mock controls (Fig. 1A). These results indicate that *SlATL2* responds to both pathogens and defense-associated hormones, highlighting its potential involvement in the plant’s defense mechanisms.

To evaluate the E3 ligase function of SlATL2, we conducted *in vitro* ubiquitination assays. After incubating SlATL2 with E1, E2, and ubiquitin, a significant accumulation of polyubiquitinated proteins was observed (Fig. 1B, lane 6), indicating that SlATL2 possesses E3 ligase activity and undergoes self-ubiquitination. To verify the importance of specific residues within the RING domain, we generated a mutant, SlATL2^C136H138A^, where cysteine 136 and histidine 138, key conserved residues, were substituted with alanines (Supplementary Data Fig. S1B), based on the functional characterization of the ATL proteins in rice [22]. *In vitro* assays showed that this mutant lacked E3 ligase activity (Fig. 1B, lane 5). These findings confirm that SlATL2 functions as an active E3 ligase and that its activity depends on an intact RING domain.

We further analyzed the subcellular localization of this protein using a chimeric SlATL2-GFP construct expressed in *N. benthamiana* leaves, in combination with the plasma membrane marker CD3–1007, a fusion of mCherry protein and AtPIP2A [52]. As shown in Fig. 1C, the fluorescence signal of SlATL2-GFP was primarily concentrated at the plasma membrane and colocalized with AtPIP2A-mCherry. Additionally, the consistent localization of SlATL2 at the plasma membrane following flg22 exposure (Fig. 1D) suggests that its subcellular localization is stable and unaffected by flg22 treatment. *Arabidopsis* AtFLS2, included as a positive control, shifts from the cell membrane to intracellular vesicles upon flg22 treatment, confirming the effectiveness of the stimulus. These findings suggest that SlATL2 functions as a plasma membrane-associated E3 ubiquitin ligase.

### SlATL2 negatively regulates immune responses against *Pst* DC3000

**Figure 2:**
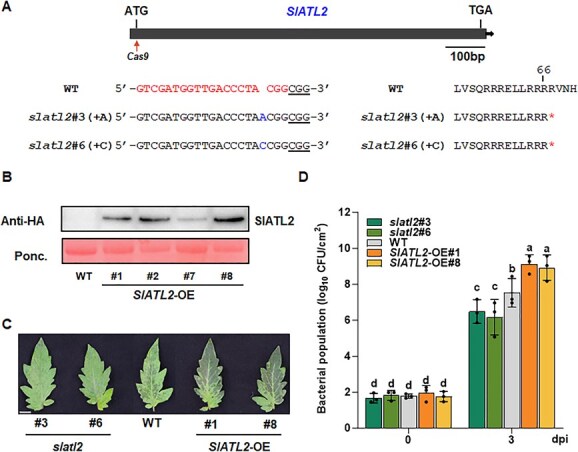
SlATL2 functions as a negative regulator of tomato immunity against *Pst* DC3000. (A) The schematic shows sgRNA target sites (indicated by the arrow) in the *SlATL2* gene and sequence details for two alleles, *slatl2*#3 and *slatl2*#6, from CRISPR/Cas9-edited T2 mutants. Nucleotide changes are marked in blue, with both alleles containing a premature stop codon at the 67th amino acid position. (B) SlATL2 overexpression lines were identified using immunoblotting. Ponceau S staining served as a loading control for protein normalization. (C) Representative images of *Pst* DC3000-caused disease symptoms in leaves of *slatl2*, *SlATL2*-OE, and WT plants at 3 dpi. Inoculation with *Pst* DC3000 was performed using vacuum infiltration. Bars = 20 mm. (D) Bacterial growth in inoculated leaves of *slatl2, SlATL2*-OE, and WT plants. Data were shown as means ± SD; *n* = 3 (D). Statistically significant differences are denoted by different letters, as determined by two-way ANOVA followed by Tukey’s test (*P* < 0.05)

To functionally characterize the function of *SlATL2* in pathogen resistance, we generated two mutants in the tomato cultivar Ailsa Craig using CRISPR-Cas9 mediated genome editing. Two mutant lines were identified: *slatl2*#3 (1-bp insertion) and *slatl2*#6 (1-bp insertion) (Fig. 2A). We additionally generated *SlATL2-HA*-overexpressing (OE) lines in the same cultivar. Two overexpression lines, *SlATL2*-OE#1 and *SlATL2*-OE#8, were used in all experiments due to their high constitutive expression levels of SlALT2 (Fig. 2B). Under greenhouse conditions, no significant growth or developmental effects were observed in either the *slatl2* or *SlATL2*-OE lines (Supplementary Data Fig. S2). Next, we characterized the response of *slatl2*, SlATL2-OE, and WT plants to *Pst* DC3000 infection. The results revealed that lesions in leaves of *sltal2* plants were smaller and less pronounced compared to those of wild-type (WT) plants (Fig. 2C). At three days postinoculation (dpi), the bacterial growth in the inoculated leaves of *slatl2* plants was significantly reduced, showing a 10∼20-fold of decrease compared to WT plants (Fig. 2D). In contrast, *SlATL2*-OE plants exhibited more pronounced disease symptoms than WT plants, characterized by larger chlorotic lesions (Fig. 2C). The bacterial population in inoculated leaves of the *SlATL2*-OE plants showed an 11.2-fold increase compared to WT plants (Fig. 2D). These findings indicate that overexpression of *SlATL2* enhances bacterial growth and disease severity, while its suppression reduces both. This suggests that SlATL2 negatively regulates tomato resistance to *Pst* DC3000.

**Figure 3:**
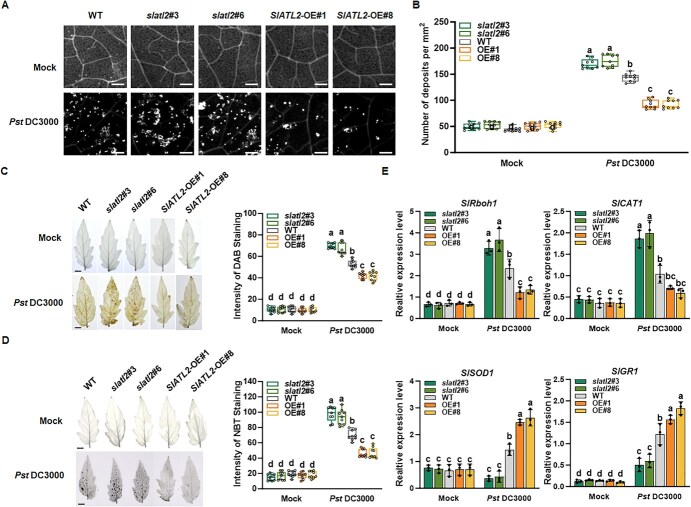
SlATL2 is involved in *Pst* DC3000-induced callose accumulation and ROS production. (A) Representative images of callose accumulation. The *slatl2*, *SlATL2*-OE, and WT plants were sprayed with *Pst* DC3000 for inoculation. Leaf samples were harvested at 24 hpi for detection. Bars = 100 μm. (B) Quantification of callose accumulation using ImageJ software, based on images shown in (A). Statistically significant differences are denoted by different letters, as determined by two-way ANOVA followed by Tukey’s test (*P* < 0.05). (C) H_2_O_2_ accumulation. Plant treatments were as described in (A). Data visualization and statistical analysis for DAB staining were performed as described in (B). Bars = 5 mm. (D) Accumulation of superoxide anion. Plant treatments were as described in (A). Data visualization and statistical analysis for NBT staining were performed as described in (B). (E) Expression of ROS homeostasis-related genes before and after infection with *Pst* DC3000. Gene expression was measured by qPCR, with *SlActin* used as the reference gene. Data were shown as means ± SD. Statistically significant differences are denoted by different letters, as determined by two-way ANOVA followed by Tukey’s test (*P* < 0.05); *n* = 9 (B-D) and *n* = 3 (E)

We further assessed callose deposition following infection with *Pst* DC3000 infection, a well-known defense response against pathogens, in *slatl2* and *SlATL2-OE* plants compared to WT plants. In mock-inoculated plants, there was no significant callose deposition, and no differences were observed among *slatl2*, *SlATL2*-OE, and WT plants (Fig. 3A and B). However, upon infection with *Pst* DC3000, callose deposition was markedly induced in cells surrounding the infection sites across all genotypes (Fig. 3A and B). Notably, *slatl2* plants showed significantly higher levels of callose deposition compared with WT plants (Fig. 3A and B). These findings suggest that silencing *SlATL2* enhances callose deposition in response to *Pst* DC3000 infection.

Reactive oxygen species (ROS) are known to mediate plant resistance to biotrophic fungal pathogens like *Pst* DC3000 [53]. Therefore, to further characterize SlATL2 function in pathogen responses, we measured ROS production in WT, *slatl2*, and *SlATL2*-OE plants following infection. In mock-inoculated plants, no pronounced differences were detected in the levels of H_2_O_2_ and superoxide anion among *slatl2*, *SLATL2*-OE, and WT plants (Fig. 3C and D), indicating that knockout or overexpression of *SlATL2* does not affect ROS accumulation. However, at 24 hpi with *Pst* DC3000, both H_2_O_2_ and superoxide anion levels significantly increased in the leaves of *slatl2*, *SlATL2*-OE, and WT plants compared with mock-inoculated controls (Fig. 3C and D). ROS levels were significantly elevated in *slatl2* plants than in WT plants, while *SlATL2*-OE plants exhibited noticeably lower ROS levels than WT (Fig. 3C and D). The transcript levels of genes associated with ROS homeostasis were also analyzed. The transcript levels of the ROS-generating gene *SlRboh1* and some ROS-scavenging genes, such as *SlCAT1* (catalase), *SlSOD1* (superoxide dismutase), and *SlGR1* (glutathione reductase), were similar among *slatl2*, SlATL2-OE, and WT mock-inoculated plants (Fig. 3E). Following inoculation with *Pst* DC3000, two distinct expression patterns were observed. Twenty-four hpi, *SlRboh1* and *SlCAT1* expression levels were significantly elevated in *slatl2* plants compared to WT, while *SlSOD1* and *SlGR1* levels were markedly reduced. Conversely, *SlATL2*-OE plants showed the opposite expression pattern (Fig. 3E). These findings suggest that knocking out *SlATL2* enhances ROS production and accumulation, whereas overexpressing *SlATL2* reduces ROS levels, supporting its role as a suppressor of pathogen resistance to *Pst* DC3000.

### SlATL2 modulates *Pst* DC3000-induced JA- and SA-dependent gene expression

**Figure 4:**
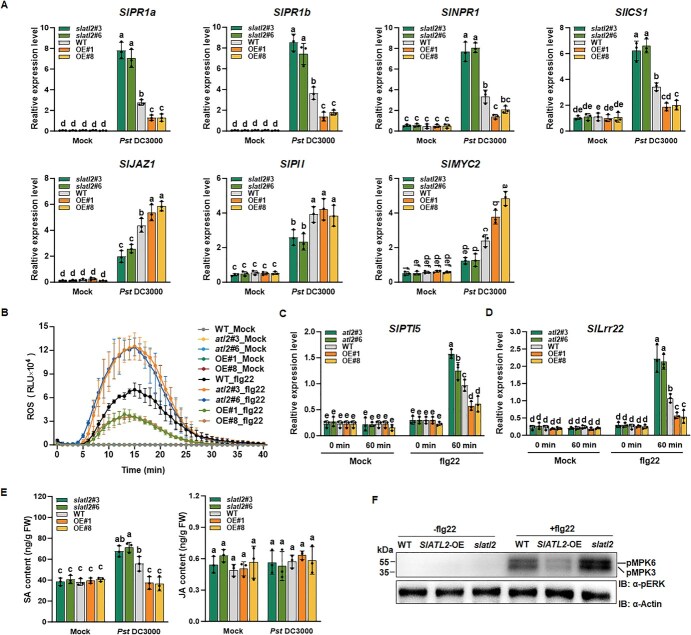
SlATL2 negatively regulates *Pst* DC3000-mediated expression of immune-related genes and flg22-induced pattern-triggered immunity (PTI). (A) Expression profiling of immune-related genes in *slatl2*, *SlATL2*-OE, and WT plants inoculated with *Pst* DC3000. Samples were harvested at 24 hpi, and gene expression was analyzed by qPCR using *SlActin* as the reference gene. (B) Flg22-induced reactive oxygen species (ROS) burst in WT, *slatl2*, and *SlATL2*-OE plants. (C, D) Changes in gene expression of *SlPTI5* (C) and *SlLRR22* (D) in *slatl2* and *SlATL2*-OE plants in response to flg22 treatment. Leaf discs were treated with flg22 (100 nM) or water, and the chemiluminescent signal was monitored immediately. *SlActin* was used as the reference gene. (E) salicylic acid (SA) and Jasmonic acid (JA) contents. (F) Abundance of phosphorylated MAPK. WT, *slatl2*, and *SlATL2*-OE plants were treated with water or 1 μM flg22 (10 min), and total proteins were probed with anti-p44/42 MAPK antibody to detect activated MPK3/6 (upper panel) and with anti-actin antibody for equal protein loading. Data were shown as means ± SD; *n* = 3 (A-E). Statistically significant differences are denoted by different letters, as determined by two-way ANOVA followed by Tukey’s test (*P* < 0.05)

To investigate the signal transduction pathways linked to SlATL2’s function in disease resistance to *Pst* DC3000, we compared the transcript levels of crucial genes engaged in JA- and SA-mediated signal transduction in *slatl2*, *SlATL2*-OE, and WT plants, both prior to and following infection with *Pst* DC3000. We selected four genes related to SA biosynthesis and signaling, such as *SlPR1a, SlPR1b*, *SlNPR1*, and *SlICS1*, and three genes involved in JA-mediated signaling, including *SlJAZ1*, *SlPI I*, and *SlMYC2* [54]. In the absence of infection, the transcript levels of these defense-related genes in both *SlATL2-OE* and *slatl2* plants were comparable to those in WT plants (Fig. 4A), suggesting that overexpression or knockout of *SlATL2* does not influence the basal defense response. However, at 48 hpi with *Pst* DC3000, SA-related gene expression was significantly increased in *slatl2* plants, while it decreased in *SlATL2*-OE plants compared to WT (Fig. 4A). In contrast, the expression patterns of JA-related genes were opposite in *slatl2* and *SlATL2-OE* plants. Despite differences in *SlATL2* expression, *SlPII* levels remained stable in WT and *SlATL2*-OE plants, potentially due to cross-talk between the SA and JA pathways stabilizing its expression, while its downregulation in *slatl2* plants suggests additional regulatory inputs under altered SA signaling. These findings underscore the intricate cross-talk between the SA and JA pathways, with SlATL2 modulating this dynamic to regulate defense and signaling gene expression upon *Pst* DC3000 infection, emphasizing its pivotal role in tomato immunity.

### SlATL2 negatively regulates flg22-induced PTI responses

To examine the role of SlATL2 in PTI, we analyzed the flg22-induced ROS burst and evaluated the transcript levels of PTI marker genes in both *slatl2* and *SlATL2-OE* plants. As expected, no ROS burst or changes in *SlPTI5* and *SlLRR22* expression were observed in mock-treated *slatl2*, *SlATL2-OE,* or WT plants (Fig. 4B-D). In response to flg22 treatment, *slatl2* plants exhibited a significantly larger ROS burst, peaking three times higher than WT, while *SlATL2*-OE plants displayed a reduced ROS burst, 1.5 times lower than WT at its peak (Fig. 4B). Additionally, *SlPTI5* and *SlLRR22* expression levels elevated by 1.2–1.6-fold and 2.0–2.1-fold, respectively, in *slatl2* plants, whereas in *SlATL2-OE* plants were reduced by 1.6–1.7-fold and 1.9–2.0-fold, compared with WT plants (Fig. 4C and D). The data demonstrate that SlATL2 suppresses flg22-induced PTI signaling in tomato.

To investigate the role of hormonal signaling, we measured SA and JA levels before and after *Pst* DC3000 infection. JA content in the leaves of *slatl2*, *SlATL2*-OE, and WT plants was similar under noninfected conditions and remained unchanged post-infection (Fig. 4E). In contrast, SA levels differed upon infection: *slatl2* plants exhibited a 1.30-fold increase, while *SlATL2*-OE and WT plants showed a 1.53-fold decrease relative to WT at 24 hpi (Fig. 4E). These results suggest that *slatl2* mutants enhances, whereas its overexpression suppresses, SA signaling and defense responses to *Pst* DC3000. Immunoblot analysis using anti-p44/42 MAPK antibodies revealed altered MAPK phosphorylation levels following flg22 treatment. Compared to WT, *slatl2* mutants showed increased phosphorylation, while *SlATL2*-OE plants displayed reduced levels (Fig. 4F). Together, these findings indicate that *slatl2* mutants enhance SA signaling and defense against *Pst* DC3000, while also exhibiting stronger immune responses to flg22 treatment.

### SlATL2 physically interacts with SlCSN5a

**Figure 5:**
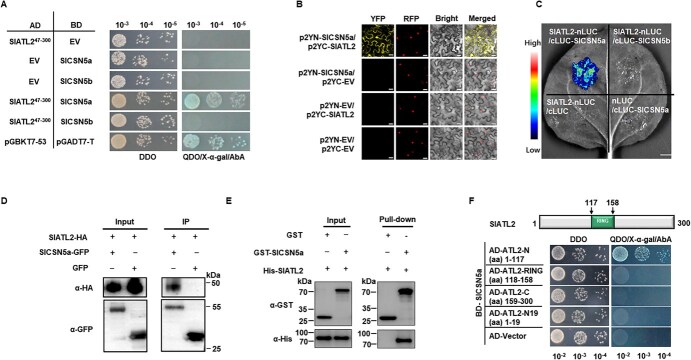
SlATL2 physically interacts with the COP9 CSN subunit SlCSN5a. (A) SlATL2 and SlCSN5a interaction by Y2H analysis. Yeast cells co-transformed with the specified vector pairs were incubated on SD/−Leu-Trp (DDO) and SD/−Leu-Trp-His-Ade (QDO)/X-α-gal/AbA plates (B) BiFC analyses of the interaction between SlATL2 and SlCSN5a. Various combinations of constructs were co-agroinfiltrated into leaves of *N. benthamiana* and the fluorescence signal was observed 2 days after agroinfiltration. Bars = 50 μm. (C) Split luciferase complementation (SLC) assays for the SlATL2-SlCSN5a interaction. SlATL2 and SlCSN5a were introduced in the NLuc and CLuc vectors, respectively. The luminescence signal was monitored at two days after infiltration. Bars = 10 mm. (D) SlATL2 and SlCSN5a interaction by co-immunoprecipitation (Co-IP) analysis. Expressed proteins were immunoprecipitated with GFP-Trap magnetic agarose beads and the associated proteins were immunoblotted with an anti-HA antibody. (E) *In vitro* pulldown assay for SlATL2 and SlCSN5a. Recombinant GST-SlCSN5a and GST were initially added to glutathione resins and incubated with His-SlATL2 for 2 h at 4°C. The eluded proteins were immunoblotted with anti-GST and anti-HIS antibodies. (F) The N terminal in SlBATL2 is essential for the SlATL2-SlCSN5a interaction. Various truncates of SlATL5 were created and assessed

RING-type E3 ligases are essential for ubiquitinating target proteins, facilitating their degradation through the 26S proteasome [55–57]. To investigate how SlATL2 regulates responses to *Pst* DC3000, a yeast two-hybrid (Y2H) assay was conducted to screen interacting proteins (Supplementary Data Table S1). This screening revealed SlCSN5a as a likely partner of SlATL2. SlCSN5a is a crucial component of the COP9 signalosome (CSN), which plays a significant role in the development and stress responses in both plants and mammals [58, 59]. Similar to *Arabidopsis*, tomato also has two CSN5 subunits, CSN5a and CSN5b. In *Arabidopsis*, these subunits are incorporated into separate CSN complexes *in vivo*, with varying abundance, where CSN5a is the more dominant subunit [29, 46]. Given the sequence similarity, we also investigated the interaction between SlATL2 and SlCSN5b. In Y2H assays, SlATL2 interacted with SlCSN5a but not with SlCSN5b, suggesting that SlCSN5b does not participate in SlATL2-mediated resistance to *Pst* DC3000 (Fig. 5A). To verify the SlATL2-SlCSN5a interaction, we conducted bimolecular fluorescence complementation (BiFC) assays, which revealed reconstituted fluorescence exclusively in the SlATL2-p2YC/SlCSN5a-p2YN combination, indicating a specific interaction *in vivo* (Fig. 5B). This interaction was further validated through split luciferase complementation (SLC) assays and co-immunoprecipitation (Co-IP) assays in *N. benthamiana*. In the SLC assays, luciferase activity was reconstituted in leaves co-infiltrated with SlATL2-nLUC and cLUC-SlCSN5a constructs (Fig. 5C). The constructs used for BiFC and SLC were all successfully expressed, as revealed by protein gel blot analysis (Supplementary Data Fig. S3). Co-IP assays demonstrated that SlATL2-HA could be immunoprecipitated with SlCSN5a-GFP using an anti-HA antibody (Fig. 5D). Direct interaction was also supported by *in vitro* pull-down assays, where GST-SlCSN5a successfully pulled down His-SlATL2 (Fig. 5E). To pinpoint the protein domains responsible for this interaction, we tested truncated versions of SlATL2 in Y2H assays. The N-terminal region of SlATL2 is essential for its interaction with SlCSN5a. Mutants lacking the N-terminal region failed to interact with SlCSN5a, while mutants retaining this region retained the interaction capability. Earlier research has revealed that the N-terminal 19 amino acids of the *Arabidopsis* ATL family member SRAS1.1 determine its interaction with AtCSN5. Therefore, we also verified whether the N-terminal 19 amino acids of SlATL2 determine its interaction with SlCSN5a. Unfortunately, SlATL2-N19 was unable to interact with SlCSN5a (Fig. 5F). In summary, these data indicate that SlATL2 interacts with SlCSN5a in both i*n vitro* and *in vivo* contexts, with the N-terminal domain of SlATL2 being essential for facilitating this interaction.

**Figure 6:**
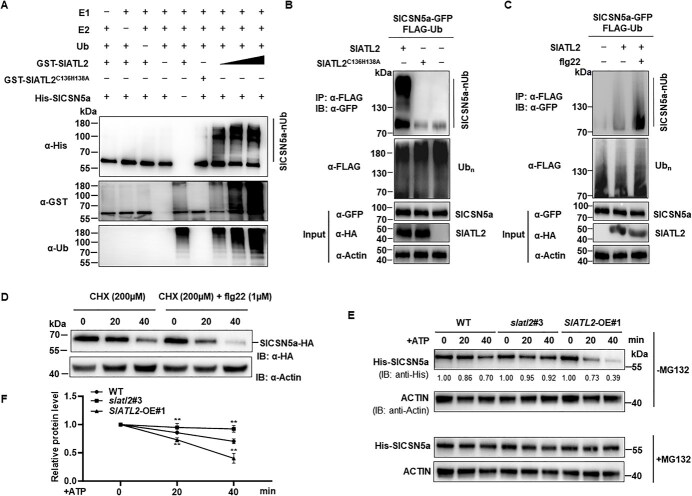
SlATL2 ubiquitinates SlCSN5a and targets it for degradation by the 26S proteasome. (A) Ubiquitination of SlCSN5a by SlATL2 *in vitro*. His-tagged SlCSN5a was co-incubated with GST-SlATL2 alongside E1, E2, and Ub. Detection of GST-SlATL2, ubiquitinated SlCSN5a, and His-SlCSN5a was performed using antibodies specific to α-GST, α-FLAG, and α-His, respectively. (B) SlATL2 ubiquitinates SlCSN5a *in vivo*. Leaves of *N. benthamiana* were co-agroinfiltrated with FLAG-Ub, SlCSN5a-GFP, and either HA-tagged SlATL2, SlATL2^C136H138A^, or a control GFP constructs. Two days after transfection, the samples were subjected to a 3-hour treatment with 2 μM MG132 and collected for analysis. The ubiquitinated SlCSN5a was immunodetected with an anti-GFP antibody after anti-FLAG immunoprecipitation. The ubiquitinated proteins were assessed using an α-FLAG antibody, while the input proteins SlATL2/SlATL2^C136H138A^ and SlCSN5a were immunodetected using α-HA and α-GFP, respectively. (C) flg22 induced SlATL2-mediated ubiquitination of SlCSN5a *in planta*. Leaves of *N. benthamiana* were co-agroinfiltrated with FLAG-Ub, SlCSN5a-GFP, and either SlATL2-HA or GFP control. Following 48 hours of agroinfiltration, the leaves were treated with 100 nM flg22 for 30 min, alongside 2 μM MG132, prior to sample collection. An anti-GFP antibody detected ubiquitinated SlCSN5a after α-FLAG immunoprecipitation. The middle panel shows total ubiquitinated proteins and the bottom panel displays input HA-tagged SlATL2 and GFP-tagged SlCSN5a proteins, as indicated by anti-FLAG, anti-HA and anti-GFP immunoblots. (D) SlCSN5a protein levels after flg22 treatment. Protoplasts were co-transfected with SlCSN5a-HA plasmid and incubated for 12 h. Cells were then challenged with flg22 with 200 μM cycloheximide or a combination of 200 μM cycloheximide +1 μM flg22 and total proteins extracted at indicated time-points were immunoblotted with α-HA antibody. (E-F) Cell-free degradation assay. Recombinant purified His-SlCSN5a was incubated with equivalent total protein extracts from 14-day-old WT, *slatl2*#3, and *SlATL2*-OE#1 seedlings with ATP present. His-SlCSN5a was immunodetected with an anti-His antibody. Panel (E) presents representative images, while panel (F) illustrates the relative protein levels. Data points are means ± SD; *n* = 3 (F). Statistically significant differences are denoted by asterisks (^**^P < 0.01, Student’s *t*-test)

### SlATL2 promotes flg22-induced degradation of SlCSN5a via the 26S proteasome

To determine if SlCSN5a is a substrate for SlATL2, we performed an *in vitro* ubiquitination assay. The results showed that SlATL2 specifically ubiquitinated SlCSN5a alongside E1, E2, and ubiquitin while the omission of these components or mutation impairing E3 ligase activity abrogated the detection of the ubiquitinated SlCSN5a (Fig. 6A). Notably, the data clearly demonstrated that increasing concentrations of SlATL2 corresponded to a proportional enhancement in the ubiquitination levels of SlCSN5a, highlighting a dose-dependent relationship. These findings confirm that SlCSN5a is a *bona fide* substrate of SlATL2. In addition, *in planta* ubiquitination assays revealed that SlCSN5a undergoes ubiquitination by SlATL2, as demonstrated by the appearance of a ladder-like smear band corresponding to SlCSN5a following immunoblot analysis with anti-GFP antibodies, followed by immunoprecipitation with anti-FLAG antibodies (Fig. 6B). Even in the absence of SlATL2, a faint ubiquitination signal was detectable, potentially due to an ATL2 homolog in *N. benthamiana*. Mutations in the conserved E2-binding cysteine and histidine residues in SlATL2 to alanine (C136H138A) significantly reduced SlCSN5a ubiquitination (Fig. 6B). Moreover, treatment with flg22 enhanced the ubiquitination of SlCSN5a by SlATL2, as evidenced by the more pronounced ladder-like smear pattern (Fig. 6C). To further address whether flg22 induces CSN5a degradation, we expressed the SlCSN5a-HA construct in protoplasts from WT tomato plants and treated them with flg22. The results showed that flg22 treatment significantly accelerated the degradation of SlCSN5a compared to untreated controls, providing additional evidence that flg22 can indeed induce SlCSN5a degradation (Fig. 6D). Additionally, we performed another Co-IP experiment to analyze whether flg22 treatment facilitates the interaction between SlATL2 and SlCSN5a (Supplementary Data Fig. S4). However, we did not observe any enhancement of their interactions upon flg22 treatment. These findings suggest that the increased ubiquitination and subsequent degradation of SlCSN5a are likely due to the elevated expression levels of SlATL2 following *Pst* DC3000 infection, as previously concluded, rather than through an enhancement of their interaction. Whether flg22 treatment triggers the autoubiquitination of SlATL2 requires further investigation.

We performed cell-free degradation assays to assess whether SlATL2 facilitates the degradation of SlCSN5a. His-SlCSN5a protein was incubated with equivalent total protein extracts from WT, *slatl2*, and *SlATL2*-OE seedlings in the company of ATP. Immunoblot analysis revealed that His-SlCSN5a degraded over time in all samples, but this degradation was significantly slower in the *slatl2* mutant and accelerated in the *SlATL2*-OE plants (Fig. 6E and F). Moreover, the degradation of SlCSN5a was abolished by MG132, a 26S proteasome inhibitor (Fig. 6E), supporting the previous findings that SlCSN5a is targeted for degradation through the 26S proteasome pathway [60]. Thus, the above findings indicate that SlATL2 mediates the ubiquitination of SlCSN5a, leading to its degradation, and flg22 can indeed accelerate this process, further enhancing the degradation of SlCSN5a.

### The role of SlCSN5a and its relationship with SlATL2

**Figure 7:**
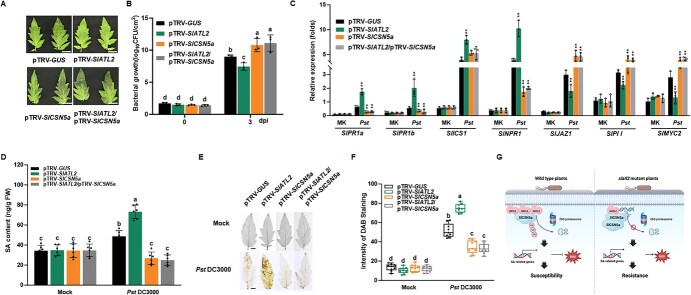
Enhanced *Pst* DC3000 resistance of *SlATL2*/*SlCSN5a*-silenced tomato plants. (A) Representative symptoms and (B) bacterial populations in leaves 3 dpi with *Pst* DC3000 in VIGS plants targeting the indicated genes. (C) Expression profiling of immune-related genes. Leaves were harvested at 24 hpi, and gene expression was analyzed by qPCR using *SlActin* as the reference gene. (D) Salicylic acid (SA) contents. (E-F) Accumulation of H_2_O_2_. Bars = 5 mm. DAB staining intensity, as determined with ImageJ software. Data were shown as means ± SD; *n* = 3 (B, C, D, F). Statistically significant differences are indicated by asterisks (^*^P < 0.05, ^**^P < 0.01, and ^***^P < 0.001, Student’s *t*-test) (C). Statistically significant differences are denoted by different letters, as determined by two-way ANOVA followed by Tukey’s test (*P* < 0.05) (B, D, F). (F) Proposed working model of SlATL2 function in tomato immunity. Upon infection with *Pst* DC3000, in WT plants, SlATL2 induces the degradation of SlCSN5a, suppressing the SA signaling pathway and ROS production, leading to susceptibility to *Pst* DC3000. In contrast, the *slatl2* mutant accumulates SlCSN5a, which activates the SA pathway and increases ROS, conferring resistance to the pathogen

Since SlATL2 ubiquitinates SlCSN5a, we next investigated the function of SlCSN5a in tomato disease resistance against *Pst* DC3000. We utilized virus-induced gene silencing (VIGS) to knock down *SlCSN5a* and *SlATL2* in tomato plants, followed by inoculation with *Pst* DC3000. To address the high-sequence similarity between *SlCSN5a* and *SlCSN5b* [*61*], we incorporated a 138 bp untranslated region (UTR) sequence of *SlCSN5a* into the *SlCSN5a* VIGS fragment to improve silencing specificity. Analysis of gene silencing specificity showed that although *SlCSN5b* expression was also somewhat reduced, the silencing of *SlCSN5a* was significantly more efficient (Supplementary Data Fig. S5). For subsequent experiments, we selected plants in which *SlCSN5b* expression was either unaffected or minimally affected, ensuring that the observed effects were primarily due to *SlCSN5a* silencing. At three dpi, plants silenced in *SlCSN5a* showed more pronounced necrotic lesions on leaves compared to control plants, which were silenced using a pTRV-*GUS* construct (Fig. 7A). Additionally, *SlCSN5a* silenced plants harbored a lower bacterial population in the infected leaves compared to controls (Fig. 7B). These findings suggest that silencing *SlCSN5a* enhances susceptibility to *Pst* DC3000.

To investigate the genetic interaction between SlCSN5a and SlATL2 in the immune response to *Pst* DC3000, we co-silenced these two genes in tomato plants using VIGS. The expression levels of *SlCSN5a* and *SlATL2* in the *SlCSN5a*, *SlATL2*, and *SlCSN5a*/ *SlATL2* silenced plants were reduced to 35%–40% of those in control plants (Supplementary Data Fig. S5). We then evaluated the changes in resistance of these tomato plants to *Pst* DC3000. In our experiments, plants co-silenced in *SlCSN5a*/*SlATL2* developed larger and denser necrotic lesions on their leaves compared to the controls, with a disease severity comparable to the plants silenced in *SlCSN5a* alone. (Fig. 7A). At three dpi, the bacterial population in the leaves of co-silenced SlCSN5a/SlATL2 plants was approximately 12 to 15 times higher than in the control plants (Fig. 7B). Based on these findings, we determined that plants co-silenced in *SlCSN5a* and *SlATL2* show increased susceptibility to *Pst* DC3000, which contrasts with the phenotypes of *SlATL2* silenced plants but phenocopies the response of *SlCSN5a* silenced plants (Fig. 7A and B). This suggests that SlATL2 functions upstream of SlCSN5a.

To determine whether the silencing of *SlCSN5a*, *SlATL2*, or their co-silencing influences the *Pst* DC3000-triggered defense response and the balance of ROS, we first assessed the expression profile of key signaling and defense-related genes in VIGS silenced plants. In mock-inoculated plants, no notable expression differences were detected in the genes associated with SA and JA signaling (Fig. 7C). However, 24 hours after infection, there was a substantial upregulation of the SA synthesis gene *ICS1*, the regulatory gene *SlNPR1*, and the defense-related genes *SlPR1a* and *SlPR1b* in VIGS plants (Fig. 7C). Notably, *SlATL2*-silenced plants exhibited higher gene expression than control plants, but lower than that observed in plants silenced for *SlCSN5a* or co-silenced in *SlATL2* and *SlCSN5a* (Fig. 7C). Consistent with these transcriptional patterns, we found that SA levels were significantly increased in *SlATL2*-silenced plants relative to WT, whereas SA content in *SlCSN5a*- and *SlATL2*/*SlCSN5a*-co-silenced plants was reduced compared to WT (Fig. 7D), supporting the notion that these genetic modifications alter SA-dependent immune responses. In addition to SA-related genes, we also analyzed the expression of JA pathway genes. We found that JA-related genes, such as *SlJAZ1*, *SlPII*, and *SlMYC2*, were downregulated in *SlATL2*-silenced plants compared to the controls, while their expression was upregulated in plants silenced for *SlCSN5a* or co-silenced for *SlATL2* and *SlCSN5a*. These findings suggest that the silencing of *SlATL2* and its interaction with SlCSN5a significantly affect the balance between SA and JA signaling pathways during the plant immune response to *Pst* DC3000. Specifically, *SlATL2* silencing enhances the SA signaling pathway and associated defense responses, whereas silencing SlCSN5a or their co-silencing suppresses these responses. These results indicate that, unlike SlATL2, SlCSN5a positively contributes to immunity against *Pst* DC3000.

Additionally, we investigated whether silencing *SlCSN5a*, *SlATL2*, or their co-silencing affects ROS homeostasis in tomato plants upon *Pst* DC3000 infection. In mock-inoculated tomato plants, no obvious differences in H₂O₂ levels were detected among VIGS and control plants (Fig. 7E and F). However, 24 hours post-inoculation (hpi), there was a substantial rise in H₂O₂ levels in all VIGS plants (Fig. 7E and F). ROS levels were significantly elevated in *SlATL2*-silenced plants relative to pTRV-*GUS* controls, while ROS accumulation was significantly reduced in *SlCSN5a* and *SlCSN5a/SlATL2* silenced plants (Fig. 7E and F). These results suggest that silencing *SlCSN5a* or co-silencing *SlATL2* and *SlCSN5a* dampens both SA-mediated defense responses and ROS production following *Pst* DC3000 infection.

## Discussion

Recent research has underscored the critical role of ATL-type E3 ubiquitin ligases in plant immunity [62]. In this study, we demonstrated that SlATL2 is crucial for resistance to *Pst* DC3000. Genetic alterations in this gene enhanced resistance in knockout lines and reduced it in overexpression lines (Fig. 2), while also affecting the expression of defense genes triggered by *Pst* DC3000 (Fig. 4A). Additionally, we established that SlATL2 contributes to PTI by analyzing flg22-triggered ROS bursts and PTI marker gene expression (Fig. 4). To investigate the underlying molecular mechanisms, we conducted a Y2H screen, identifying SlCSN5a, a COP9 signalosome subunit, as an interacting partner of SlATL2. This interaction was confirmed through BiFC, SLC, Co-IP, and *in vitro* pull-down assays (Fig. 5). Both *in vitro* and *in vivo* ubiquitination assays revealed that SlATL2 acts as an E3 ligase, mediating the polyubiquitination and degradation of SlCSN5a (Fig. 6). The knockout of *SlATL2* led to decreased degradation of SlCSN5a, which was induced by flg22 and dependent on the proteasome (Fig. 6). Additionally, silencing *SlCSN5a* or co-silencing both *SlATL2* and *SlCSN5a* resulted in heightened susceptibility to *Pst* DC3000, highlighting SlCSN5a as a positive regulator of defense that operates downstream of SlATL2. (Fig. 7). Overall, these findings suggest that the RING E3 ubiquitin ligase SlATL2 negatively regulates immune responses by facilitating the polyubiquitination and degradation of SlCSN5a.

The resistance to (hemi)biotrophic pathogens like *Pst* DC3000 is well established to be governed via the SA signaling [63–65]. In *slatl2* mutant plants, the transcript levels of the SA biosynthetic gene *ICS1*, the SA signaling regulator *SlNPR1*, and the SA-responsive defense genes *SlPR1a* and *SlPR1b* were elevated in response to *Pst* DC3000, compared to WT controls. Conversely, in *SlATL2*-OE overexpression lines, their expression was moderately suppressed (Fig. 4A). The expression of the JA signaling regulators *SlJAZ1* and *SlMYC2*, along with the JA-responsive defense gene *SlPII*, showed an opposite expression trend in both *SlATL2*-OE and *slatl2* plants following *Pst* DC3000 infection (Fig. 4A). Silencing *SlCSN5a* and co-silencing *SlATL2* and *SlCSN5a* plants also exhibited similar expression patterns for SA and JA-related genes. These contrasting expression profiles suggest that the SA signaling pathway is essential for the role of SlATL2 in resistance to *Pst* DC3000, while SA signaling appears to antagonize JA signaling to some extent. This finding aligns with earlier reports that *Arabidopsis* ATL2, ATL31, and ATL6 positively influence SA-mediated immune gene expression [17, 21], but diverges from observations that LeATL6 plays a positive role in JA signaling without affecting the SA pathway in response to fungal elicitors [28].

In the early stages of PTI, the ROS burst plays a crucial role in disease resistance [66, 67]. Our experiments showed that *slatl2* plants exhibited an enhanced flg22-induced ROS burst, whereas *SlATL2*-OE plants displayed a delayed and significantly suppressed response (Fig. 4B). Additionally, the PTI marker genes *SlPTI5* and *SlLRR22* expression levels were elevated in *slatl2* plants but diminished in *SlATL2*-OE plants (Fig. 4C and D). These results suggest that SlATL2 plays a negative role in the regulation of tomato PTI. This conclusion is further supported by the phosphorylation status of MAPKs, as *slatl2* plants displayed higher MPK3/6 activation, whereas *SlATL2*-OE plants showed reduced MPK3/6 activation compared to WT following flg22 treatment (Fig. 4F). Notably, both *SlATL2*-OE#1 and *SlATL2*-OE#8 lines exhibited defects in both ROS production and MAPK activation, prompting the question of whether SlATL2 functions as part of the PRR complex. To explore this possibility, we used BiFC assays to test for potential interactions between SlATL2 and flg22-related PRR complex components SlFLS2 and SlBAK1. However, the results showed that SlATL2 does not interact with either SlFLS2 or SlBAK1, suggesting that SlATL2 does not exert its function through direct regulation of the PRR complex (Supplementary Data Fig. S6). Previous reports indicate that overexpression of *AtATL31* and *AtATL6* resulted in increased resistance to *Pst* DC3000, suggesting that AtATL31 and AtATL6 act as positive regulators of resistance to pathogen infection [21]. The contrasting roles of SlATL2 compared to AtATL31 and AtATL6 may stem from their belonging to different clades, indicating functional divergence through evolutionary processes (Supplementary Data Fig. S7).

Consistent with previous findings in the tomato-*Pst* DC3000 interaction [68], we also observed significant callose accumulation at infection sites following *Pst* DC3000 inoculation (Fig. 3A). Moreover, we found that *slatl2* mutants exhibited greater accumulation of callose compared to WT controls (Fig. 3A). This observation aligns with earlier reports in *Arabidopsis*, where *atl31* and *atl6* mutant plants showed increased callose deposition in reaction to flg22 treatment [21]. Nonetheless, the role of callose in disease resistance is multifaceted [69]; for instance, reduced callose accumulation did not significantly impact resistance to *Pst* DC3000 in *Arabidopsis*, highlighting the complexity of this relationship [70]. Nevertheless, our findings suggest that SlATL2 regulates callose deposition at infection sites, reinforcing the notion that callose accumulation contributes to the defense against *Pst* DC3000 in tomato [68].

ROS production is a fundamental immune response shared by both plants and animals. ROS functions as an antimicrobial agent while also serving as a crucial signaling molecule in plant innate immunity [71]. In this research, we discovered that *slatl2* plants exhibited significantly higher levels of H₂O₂ and superoxide anions, while their levels were lower in *SlATL2*-OE plants compared to WT plants following infection with *Pst* DC3000 (Fig. 3C and D). Notably, in the absence of infection, *slatl2* and *SlATL2*-OE plants showed no significant differences in H₂O₂ and superoxide anion accumulation compared with the WT plants (Fig. 3C and D). These findings suggest that the knockout of *SlATL2* may disrupt the regulation of ROS production and scavenging during pathogen attack. Supporting this, we noted increased expression of *SlRboh1*, which is associated with heightened H₂O₂ levels [72], in *slatl2* plants post-infection, while the transcript level of ROS-scavenging genes *SlSOD1* and *SlGR1* decreased (Fig. 3E). In contrast, the opposite expression pattern was observed in *SlATL2*-OE plants (Fig. 3E). The increased expression of *SlCAT1* in *slatl2* plants after *Pst* DC3000 infection likely results from feedback regulation due to the excessive ROS accumulation within the cells (Fig. 3E). Knockout of *SlATL2* seems to enhance ROS accumulation induced by *Pst* DC3000 by disrupting the ROS homeostasis-related gene expression, which may contribute to increased resistance against the pathogen. Tomato resistance to the necrotrophic fungus *B. cinerea* is also closely linked to ROS accumulation [65]. To investigate whether SlATL2 also regulates resistance to necrotrophic fungal pathogens, we examined the disease phenotype of the *slatl2* plants after inoculation with *B. cinerea* using a detached leaf inoculation assay (Supplementary Data Fig. S8). The results demonstrated that *slatl2* mutants were significantly more susceptible to *B. cinerea* compared to WT plants as evidenced by both lesion size and fungal growth assays, showing a phenotype similar to that of the homologous *atatl2* mutants in *Arabidopsis thaliana* [*16*]. This finding highlights a positive regulatory role of SlATL2 in tomato resistance to *B. cinerea*. Together, these results reveal that SlATL2 functions in contrasting ways in tomato defense, positively regulating resistance to the fungal pathogen *B. cinerea* while negatively regulating resistance to the bacterial pathogen *Pst DC3000*.

To further delineate the mechanism by which SlATL2 contributes to resistance against *Pst* DC3000, we screened a tomato cDNA yeast two-hybrid (Y2H) library, derived from *Pst* DC3000-infected plants, using SlATL2 as the bait (Supplementary Data Table S1). This screening identified SlCSN5a as a potential interacting partner. This interaction was further confirmed by Y2H analysis (Fig. 5A), SLC (Fig. 5C), Co-IP (Fig. 5D), and GST pull-down assays. Moreover, the two proteins were localized in the nucleus and the plasma membrane, as demonstrated by the *in vivo* BiFC system (Fig. 5B). Additionally, we conducted *in planta* co-localization assays for SlCSN5a and SlATL2, and the results showed that both proteins co-localize at the plasma membrane (Supplementary Data Fig. S9). While SlCSN5a is localized in both the plasma membrane and the nucleus, it is possible that under specific conditions, SlATL2 may translocate from the plasma membrane to the nucleus and interact with SlCSN5a there. However, no co-localization of the two proteins was observed in the nucleus under the tested conditions. This co-localization at the plasma membrane supports the proposed functional interaction between SlCSN5a and SlATL2 in immune signaling at the membrane. Further characterization of this interaction demonstrated that it occurs via the N-terminal region of SlATL2 (Fig. 5F), rather than in its RING domain or C-terminal region. This finding aligns with observations in *Arabidopsis*, where the Salt-Responsive Alternatively Spliced gene 1 (SRAS1)/AtATL27 also interacts with CSN5a through its N-terminal region [60]. However, it contrasts with the interaction mechanisms of ATL31 with 14–3-3χ, which is regulated by the C-terminal region [73]. Moreover, phosphorylation of Ser/Thr residues in the C-terminal fragment of ATL31 affects both its stabilization and its binding to 14–3-3χ [22]. Thus, additional investigations are necessary to elucidate the regulatory mechanisms, particularly the role of phosphorylation, that dictate the targeting of the ubiquitin ligase SlATL2 to the SlCSN5a protein. While a previous study has shown that the binding of SRAS1.1, a RING-type E3 ligase gene in *Arabidopsis*, to CSN5A is likely promoted by a short N-terminal motif [60]. However, in our Y2H experiments using a truncated version of the protein, we found that the N-terminal amino acid 1–19 of SlATL2 does not interact with SlCSN5a, suggesting that this short N-terminal motif alone may not be sufficient for SlATL2-SlCSN5a interaction.

*In vitro* ubiquitination assays conducted with ubiquitin, E1, and E2 enzymes demonstrated that SlATL2 undergoes auto-ubiquitination, confirming its E3 ligase activity (Fig. 1B). Additionally, SlATL2 facilitated the ubiquitination of the COP9 signalosome subunit SlCSN5a, leading to its subsequent degradation (Fig. 6A-F). Following flg22 treatment, a noticeable increase in the ubiquitination levels of SlCSN5a was observed，a finding further supported by *in planta* degradation assays demonstrating that flg22 accelerates SlCSN5a degradation. (Fig. 6C and D). This observation is in line with findings from *Arabidopsis*, where ATL E3 ligases, such as ATL31 and ATL6, stabilize the receptor-like cytoplasmic kinase BIK1 by promoting the degradation of CPK28, thereby enhancing immune responses through increased ubiquitination following flg22 treatment [22]. Conversely, OsATL32 in rice mitigates pathogen-induced ROS accumulation by facilitating the degradation of the ROS-producing module, thereby bolstering disease resistance to *Magnaporthe oryzae*. Remarkably, OsATL32 ubiquitination decreases after chitin treatment [74]. Plants infiltrated with pTRV-*SlATL2* exhibited elevated ROS levels and enhanced expression of SA-related genes after *Pst* DC3000 infection. In contrast, pTRV-*SlCSN5a*-infiltrated plants showed reduced ROS levels in the same context (Fig. 7D and E). These results collectively suggest a direct regulatory relationship between SlATL2 and SlCSN5a, where SlATL2 targets SlCSN5a for degradation, inhibiting ROS production and SA signaling. This suggests that the suppression of the flg22-induced ROS burst by the SlATL2-SlCSN5a module may involve mechanisms beyond gene expression regulation. The COP9 signalosome (CSN), to which SlCSN5a belongs, is known to participate in diverse cellular processes, including oxidative stress regulation [29, 75]. The ubiquitination and degradation of SlCSN5a by SlATL2 may disrupt CSN function, potentially influencing the activity or stability of ROS-scavenging enzymes. While our study primarily focuses on the effect of the SlATL2-SlCSN5a module on the expression of SA-responsive defense genes, preliminary assays suggest that SlATL2 might also enhance ROS-scavenging activity under *Pst* DC3000 inoculation conditions, thereby contributing to the suppression of ROS accumulation. These findings suggest that the regulation of ROS by the SlATL2-SlCSN5a module is multifaceted, involving both transcriptional and post-translational mechanisms. Further studies are needed to elucidate the molecular basis of these interactions and their implications for plant immunity. This mechanism is further corroborated by earlier studies showing that CSN5a regulates transcription factors like HY5, which in turn influences the transcript levels of ROS-scavenging enzymes such as catalase and superoxide dismutase [76]. Consequently, CSN5a indirectly affects antioxidant enzymes like ascorbate peroxidase, catalase, and superoxide dismutase, which are essential for maintaining ROS homeostasis [77]. Previous studies have established that rice E3 ligases like SPL11, APIP6, and ATL32 modulate ROS levels and immune responses by targeting both ROS-producing regulatory factors and ROS-scavenging enzymes [74, 78, 79]. However, prior to this study, there was no direct evidence linking immunity-related E3 ligases with the degradation of COP9 signalosome subunits. Here, we propose a novel mechanism in which SlATL2 negatively regulates ROS production by targeting the photomorphogenic factor SlCSN5a for degradation.

Of note, the immune functions of ATL2 and CSN5 in tomato differ from their roles in other plants. Specifically, our phenotypic analyses using CRIPSR-Cas9-edited mutants and overexpression lines revealed a negative function of ATL2 in tomato immunity, inconsistent with the positive role of a homologous ATL2 in *N. benthamiana* [80]. We and others revealed that CSN5 positively contributes to tomato immunity against *Pst* DC3000, *Botrytis cinerea*, herbivorous insects, and root-knot nematodes [50, 81], which is inconsistent with its previously reported negative regulatory role in *Arabidopsis*, rice, wheat, and grape [47, 82–84]. Several factors may contribute to the discrepancies in ATL2 and CSN5 immune functions across different plants. Species-specific immune signaling network and functional divergence may shape the distinct roles of ATL2 and CSN5. Our observations that ATL2 ubiquitinates CSN5 for degradation (Fig. 6) and requires CSN5 for its function (Fig. 7A and B) place them within the same regulatory cascade, where ATL2 acts as a negative regulator and CSN5 as a positive regulator of immunity. Alternatively, differences in experimental systems could influence the observed phenotypic outcomes. We used stable knockout and overexpression lines in tomato, a well-characterized host of *Pst* DC3000, whereas Li *et al.* utilized a VIGS system in *N. benthamiana* [80], a species where many aspects of its interaction with *Pst* DC3000 remain unclear. Such methodological differences may impact the interpretation of the ATL2’s role in plant immunity. A similar phenomenon was observed in tomato *PORK1*, where CRISPR-Cas9 knockout lines retained full system in responsiveness [85], contradicting earlier findings based on RNAi-mediated knockdown lines [86]. Furthermore, in addition to the discovery that OsCSN5 serves as a substrate for OsPUB45 in rice, recent studies have also identified *Arabidopsis* CSN5A as a target of AtATL27 [60], suggesting that CSN5 may act as a target for different E3 ligases across plant species or serve as a substrate for multiple E3 ligases within the same species. The functional divergence of ATL2 and CSN5 in tomato highlights the complexity of its immune regulation and the need for further investigation into species-specific immune networks. It is interesting to determine whether ATL2 and CSN5 have diversified their immune roles across different plant lineages.

## Conclusion

In summary, our research demonstrates that SlATL2 plays a negative regulatory role in resistance to *Pst* DC3000 by disrupting the SlCSN5a, thereby inhibiting SA signaling and ROS production. Based on these findings, we propose a novel mechanism in plant immunity (Fig. 7G). Upon infection with *Pst* DC3000, the WT plants exhibit induction of the membrane-associated SlATL2, which facilitates the ubiquitination and subsequent degradation of SlCSN5a. This degradation suppresses the expression of genes linked to the SA signaling pathway and reduces ROS production, making WT plants more susceptible to *Pst* DC3000. In contrast, the *slatl2* mutant lacks functional SlATL2, preventing the degradation of SlCSN5a and resulting in its accumulation. The increased levels of SlCSN5a enhance the activation of the SA signaling pathway and elevate ROS production, thereby strengthening the immune response and conferring resistance to *Pst* DC3000.

## Materials and methods

### Plant cultivation, treatments, and disease assays

The experiment utilized *Solanum lycopersicum* L. cv. Ailsa Craig, grown under controlled conditions: 200 μmol m^−2^ s^−1^ light intensity, 22–24°C, 60% relative humidity, and a 14 h/10 h light–dark cycle. Pathogenicity assays and bacterial growth quantification followed standardized protocols [87].

### Subcellular localization assays

To examine SlATL2 and SlCSN5a localization, agrobacterium carrying pCAMBIA1300-SlATL2-GFP, pCAMBIA1300-SlCSN5a-GFP, or pCAMBIA1300-GFP was co-infiltrated into *N. benthamiana* with the CD3–1007 vector expressing the plasma membrane marker AtPIP2A-mCherry [52]. Following infiltration, the leaves were treated with either 100 nM flg22 (GeneScript, Piscataway, NJ, USA) or water as a control. Fluorescence from GFP and mCherry was examined at 2 dpi using Zeiss LSM780 microscope.

### Characterization of transgenic lines

The *SlATL2* CDS was subcloned into pFGC1008-HA, yielding pFGC1008-*SlATL2*-HA. CRISPR/Cas9 vectors were designed following [88] to generate *slatl2* mutants. A single guide RNA (sgRNA) targeting the first exon of *SlATL2* was selected using the CRISPR-P program and incorporated into the expression cassette. The plasmids were transformed into Ailsa Craig following [89] to generate *SlATL2*-OE and *slatl2* lines. *SlATL2*-OE lines were identified as detailed in prior research [87]. Homozygous *slatl2* mutants were identified in the T1 generation. After confirming sequencing, independent homozygous F₂ lines were selected for following study.

### VIGS assays

VIGS fragments corresponding to the *SlATL2* and *SlCSN5a* genes were subcloned into the pTRV2 vector [90], generating the plasmids pTRV-*SlATL2* and pTRV-*SlCSN5a*. Sequence for these VIGS fragments can be found in Supplementary Data Table S2. To achieve co-silencing of both genes, a mixed agrobacterium suspension containing pTRV-*SlATL2* and pTRV-*SlCSN5a* was utilized. The VIGS protocol was performed on two-week-old tomato seedlings [90]. Silencing specificity and efficiency were assessed by qRT-PCR after three weeks using primers listed in Supplementary Data Table S3.

### ROS assays

ROS assays followed the method in [91]. Leaf disks (0.2 cm^2^) were placed in water in a 96-well plate and incubated overnight. The next day, 20 mg/mL horseradish peroxidase, 200 mM luminol, and 100 nM flg22 were added. Chemiluminescence was recorded every 2 minutes for 30 minutes using a Synergy HT Microplate Reader (Biotek Instruments).

### Callose staining

Callose staining followed the method in [54]. Samples were subjected to alcoholic lactophenol at 65°C for 30 minutes, then transferred to fresh solution overnight. After rinsing with 50% ethanol and washing twice with deionized water, samples were stained with aniline blue (0.01% in 150 mM sodium phosphate buffer) for 1 hour in the dark. Callose accumulation was visualized by fluorescence microscopy and quantified using ImageJ.

### *In situ* histochemical staining/DAB and NBT staining

Histochemical staining for H₂O₂ and superoxide anion followed modified protocols [54, 92]. Leaves from inoculated tomatoes (24 hpi) were stained with 1 mg/mL DAB (pH 3.8, 8 h, dark) for H₂O₂ or 0.1% NBT (10 mM phosphate buffer, pH 7.5, 10 mM NaN₃, 1 h, RT) for superoxide detection. Chlorophyll was removed with ethanol treatment. Images were obtained under a dissecting microscope and analyzed by ImageJ.

### Quantification of SA andJA

Leaf tissue (~50 mg) was extracted in 1 mL of ethyl acetate containing 20 ng D6-JA and 5 ng D6-SA as internal standards. The samples were analyzed on a Waters Xevo TQ-S HPLC-triple quadrupole LC–MS system following a previously described method [93].

### MAPK assays

MAPK activation was assessed in two-week-old seedlings grown on MS agar. Seedlings were treated with 1 μM flg22 or water for 15 min. MAPK phosphorylation (pTEpY) was detected using phospho-p44/42 MAPK antibodies (Cell Signaling Technology). Anti-Actin antibody (Merck) served as a loading control.

### Y2H assays

The Matchmaker Gold Yeast Two-Hybrid System (Takara Bio) was used for Y2H as per the manufacturer’s recommendations. For this screening, the truncated SlATL2^51–300^ cDNA sequence, which does not include the TM-containing N-terminal region, served as the bait to screen the Y2H cDNA library. Positive colonies were first identified on SD/−Leu/−Trp/-His plates and then on SD/−Leu/−Trp/-His/−Ade/X-α-gal plates for further validation. To confirm protein–protein interactions, constructs pGBKT7-SlCSN5a or pGBKT7-SlCSN5b were co-transformed with pGADT7-SlATL2 into the yeast strain Y2HGold. Additionally, pGBKT7-SlCSN5a was co-transformed with SlATL2 deletion mutants fused to pGADT7 vectors into the yeast strain Y2HGold. The primers used in these procedures are detailed in Supplementary Table S3.

### BiFC and SLC assays

To investigate the interactions between SlATL2 and SlCSN5a, BiFC and SLC assays were performed, utilizing the methodologies described previously [87]. The CDSs for *SlATL2* and *SlCSN5a* were respectively subcloned into p2YC and p2YN vectors at the *Pac*I-*Asc*I restriction sites, resulting in the plasmids p2YN-SlCSN5a and p2YC-SlATL2. Similarly, these CDSs were inserted into pCAMBIA-35S-nLuc and pCAMBIA-35S-cLuc vectors to generate SlATL2-nLUC and cLUC-SlCSN5a constructs, respectively. The resulting fusion proteins were co-agroinfiltrated in *N. benthamiana* leaves, as previously outlined [87], alongside a nucleus-localized marker protein, RFP-H2B [94]. For the SLC assay, *N. benthamiana* leaves were treated with 0.5 mM luciferin, and luminescence was captured using a Tanon system. YFP and RFP signals in the BiFC assay were observed at 48 hpi with a Zeiss LSM780 confocal microscope. Leaves expressing the fusion proteins were harvested 48 hours after inoculation for protein expression analysis in split-LUC assays or BiFC assays. Primers for the BiFC and SLC assays are listed in Supplementary Data Table S3.

### Co-IP assay

Co-IP assays were performed following a previously established protocol [95]. In brief, the CDS of *SlCSN5a* was inserted into pCAMBIA1300-GFP to create pCAMBIA1300-GFP-SlCSN5a. pFGC1008-SlATL2-HA and pCAMBIA1300-GFP-SlCSN5a constructs were co-agroinfiltrated into *N. benthamiana* leaves. Two days post-infiltration, proteins were extracted and incubated overnight at 4°C with GFP-Trap beads (ChromoTek). After washing, the protein complexes were immunoblotted with anti-GFP (Abcam) or anti-HA antibodies (Sigma-Aldrich). The primers used in these assays are listed in Supplementary Data Table S3.

### *In vitro* pull-down assays

The GST pulldown assay followed established protocols [96]. GST-tagged SlATL2 and His-tagged SlCSN5a were produced through bacterial expression in *E. coli* Rosetta (DE3) strain, followed by affinity purification using Glutathione resin (Genscript) or Ni-NTA agarose (Qiagen) respectively. Following a 4-hour cold incubation (4°C) between either GST control or GST-SlATL2 with SlCSN5a-His, eluted proteins were detected with anti-His and anti-GST antibodies (Sigma-Aldrich).

### *In vitro* and *in vivo* ubiquitination assays

The ubiquitination experiments *in vitro* were conducted according to established protocols from reference [97]. Specifically, the CDS of SlCSN5a was engineered into the pET32a vector, whereas SlATL2 and its modified variant (SlATL2^C138H138A^) were ligated into the pGEX-4 T-3 plasmid. These recombinant vectors were subsequently expressed in *E. coli* Rosetta (DE3) cells through induction with 0.3 mM IPTG during a 20-hour incubation. Post-cultivation, bacterial cells were pelleted via centrifugation, and target proteins were affinity-purified using either Ni-NTA agarose (QIAGEN) or glutathione-coupled matrices (Genscript) in strict compliance with the manufacturers’ operational guidelines.

For the self-ubiquitination, 2 μg GST-SlATL2 or GST-SlATL2^C138H138A^ were incubated with 110 ng E1 (R&D Systems), 220 ng UbcH5a (R&D Systems) and 5 μg ubiquitin-FLAG (R&D Systems) in reaction buffer at 30°C for 3 h. Substrate ubiquitination assays were performed by incubating His-*SlATL2* with varying amounts of GST-tagged *SlCSN5a* in the same reaction buffer. Detection was performed with anti-FLAG (Sigma-Aldrich), anti-GST (GenScript), and anti-His (GenScript) antibodies, respectively.

For the *in vivo* ubiquitination assay, constructs for FLAG-Ubi, SlCSN5a-GFP, and either SlATL2-HA or SlATL2^C136H138A^-HA were agroinfiltrated into leaves of *N. benthamiana* with or without 100 nM flg22. Ubiquitinated proteins were immunoprecipitated using anti-FLAG M2 affinity resin (Sigma-Aldrich) and immunoblotted by anti-GFP (Abcam) for SlCSN5a-GFP, and anti-HA, and anti-FLAG (Sigma-Aldrich) antibodies.

### *In planta* degradation and cell-free degradation assays

*In planta* degradation assays in tomato protoplasts followed a modified protocol from [98]. Protoplasts co-transfected with SlCSN5a-HA were incubated for 12 h, then subjected with 200 μM cycloheximide either in isolation or supplemented with 1 μM flg22. Proteins were extracted at specified times and immunoblotted with α-HA and anti-Actin antibodies.

For cell-free degradation [99], equal amounts of *the* total proteins from two-week-old WT, *slatl2#*3, and *SlATL2-*OE#1 seedlings were incubated with purified His-SlCSN5a at 25°C in ATP-containing buffer, with or without MG132. Anti-His and anti-Actin antibodies were used for immunoblotting.

### RT-qPCR assays

Total RNA was isolated using VeZol reagent (Vazyme Biotech) and reverse transcribed with HiScript IV RT SuperMix for qPCR (Vazyme Biotech). The qPCR assays were carried out with SupRealQ Purple Universal SYBR qPCR Master Mix (Vazyme Biotech) on a Roche LightCycler 96 system. Relative gene expression was calculated by the 2^–△△CT^ method using *SlActin* served as the internal control. Primers are listed in Supplementary Data Table S3.

## Supplementary Material

Web_Material_uhaf078

## Data Availability

All study data are incorporated in the submitted article.
