# Supplementary material for: The E3 ubiquitin ligase SlATL2 suppresses tomato immunity by promoting SlCSN5a degradation during Pseudomonas syringae pv. tomato DC3000 infection
Source: Hortic Res. 2025 Mar 10;12(6):uhaf078. doi: 10.1093/hr/uhaf078 (PMC12038897; doi:10.1093/hr/uhaf078)
Supplement: Web_Material_uhaf078 [file web_material_uhaf078.zip › Web_Material_uhaf078.docx]

**The E3 ubiquitin ligase SlATL2 suppresses tomato immunity by promoting SlCSN5a degradation during *Pseudomonas syringae* pv*. tomato* DC3000 infection**

**Yujie Dai ^1,2,3,4^, Xiaodan Li ^1,2,3,4^, Yeling He ^1,2,3^, Liya Zhu ^1,2,3^, Yan Bi ^1,2,3^, Fengming Song ^1,2,3*^, and Dayong Li ^1,2,3*^**

**^1^** Key Laboratory of Crop Diseases and Insect Pests of Ministry of Agriculture, Institute of Biotechnology, Zhejiang University, Hangzhou 310058, P. R. China

**^2^** Zhejiang Key Laboratory of Biology and Ecological Regulation of Crop Pathogens and Insects, Institute of Biotechnology, Zhejiang University, Hangzhou 310058, P. R. China

**^3^** State Key Laboratory for Rice Biology and Breeding, Institute of Biotechnology, Zhejiang University, Hangzhou 310058, P. R. China

**^4^** Both authors contributed equally

Yujie Dai: dyjjane@163.com

Xiaodan Li: 1034720876@qq.com

Yeling He: 2214473378@qq.com

Liya Zhu: 3190101388@zju.edu.cn

Yan Bi: 1481726327@qq.com

***Correspondence:**

Dr. Dayong Li: dyli@zju.edu.cn

Tel: +86-571-88981305, Fax: +86-571-88981305

Dr. Fengming Song, Email: fmsong@zju.edu.cn

Tel: +86-571-88981305, Fax: +86-571-88981305

**Running head:** Tomato SlATL2 in Immunity to *Pst* DC3000

**
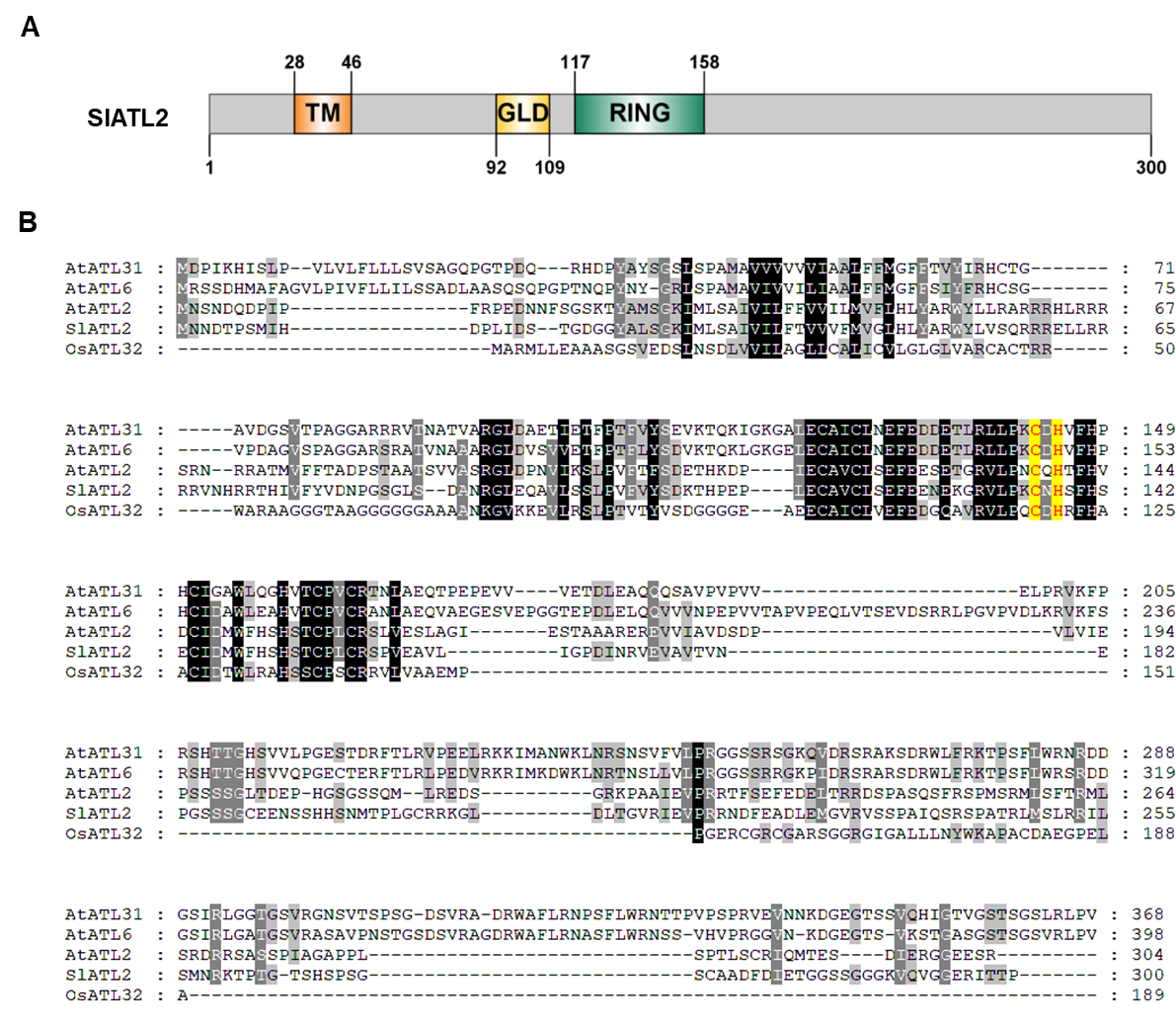
**

**Figure S1.** Sequence and domain analysis of SlATL2. (A) The predicted domains for SlATL2. TM: transmembrane domain; GLD: highly conserved motif containing Gly-Leu-Asp residues; RING: RING-H2 type zinc finger domain. (B) Alignment of SlATL2 with other ATLs from *Arabidopsis* and rice. The numbers on the right indicate amino acid positions of the ATL proteins. The enzymatically active sites are highlighted in yellow. NCBI accession numbers of ATLs are as follows: AtATL31 (NP_198094.1), AtATL6 (NP_566249.1), AtATL2 (NP_188294.1), OsATL32 (XP_015625263), SlATL2 (XP_004229261).


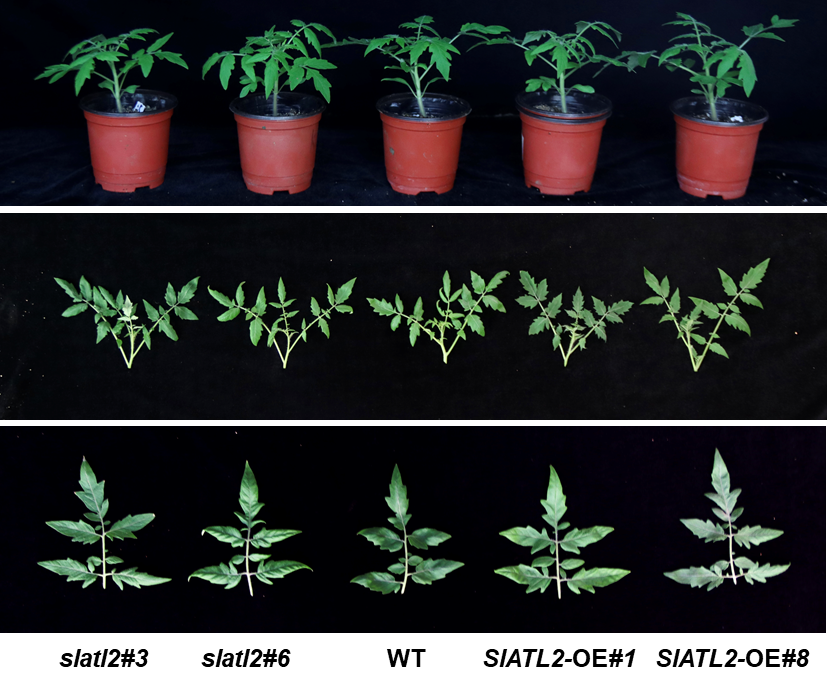


**Figure S2.** Growth phenotype of the *slatl2*, *SlATl2*-OE and WT plants.


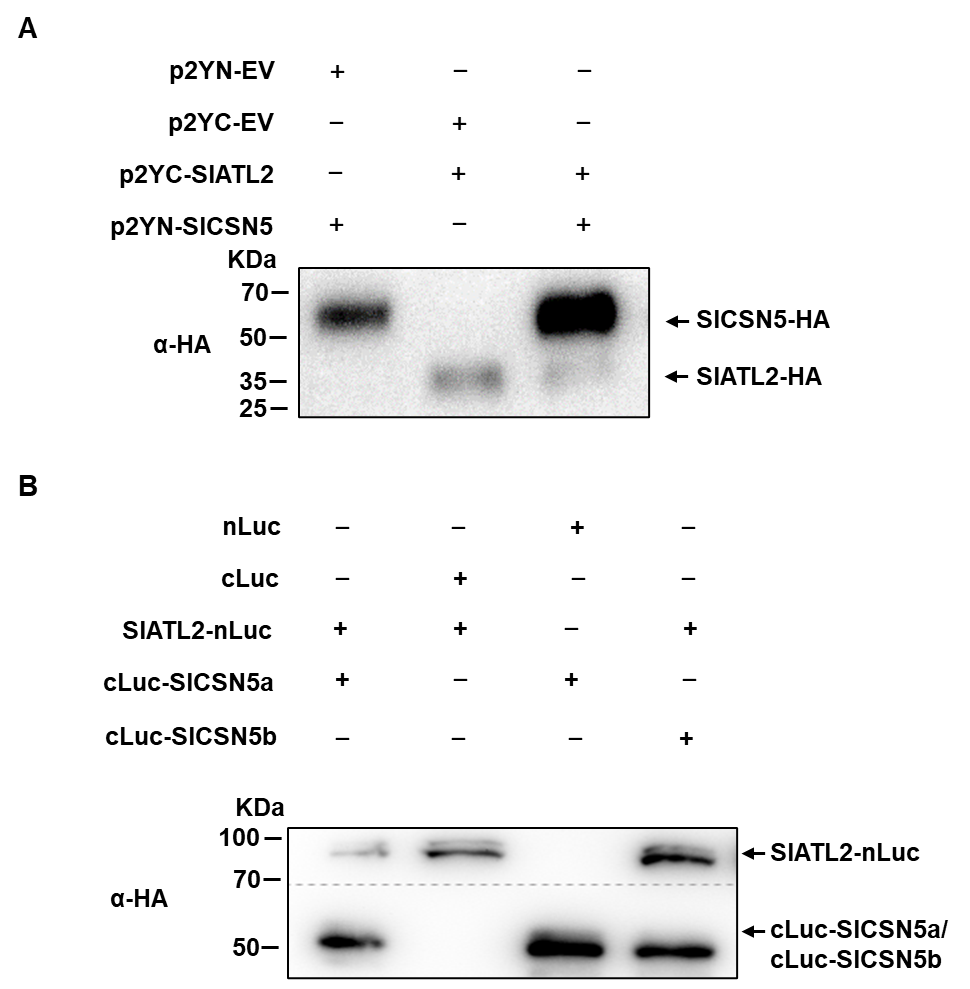


**Figure S3.** Accumulation of SlATL2, SlCSN5a and SlCSN5b in *N. benthamiana* leaves in BiFC and SLC assays. (A) Agrobacterium carrying different p2YC and p2YN pairs were infiltrated into *N. benthamiana* leaves and leaf samples at 48 h after infiltration were collected for protein extraction. Fusion proteins were determined by immunoblot analysis with anti-HA antibody. (B) Agrobacterium carrying different nLuc and cLuc pairs were infiltrated into *N. benthamiana* leaves and leaf samples at 48 h after infiltration were collected for protein extraction. Fusion proteins were determined by immunoblot analysis with anti-HA antibody. All experiments were conducted at least three times with similar results.

**
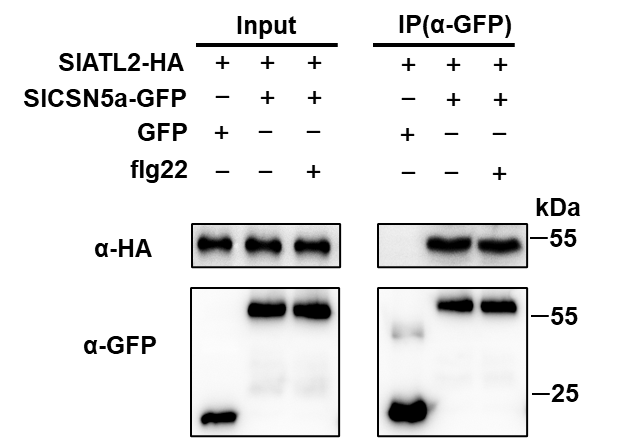
**

**Figure S4.** SlATL2 and SlCSN5a interaction after flg22 treatment. SlATL2-HA was coexpressed with SlCSN5a-GFP in *N. benthamiana*. Total proteins were extracted, immunoprecipitated with GFP-Trap magnetic beads, and immunoblotted with anti-HA or anti-GFP antibody. Plants were treated without or with 1 mM flg22 for 1 h. Three independent experiments were carried out with similar results.


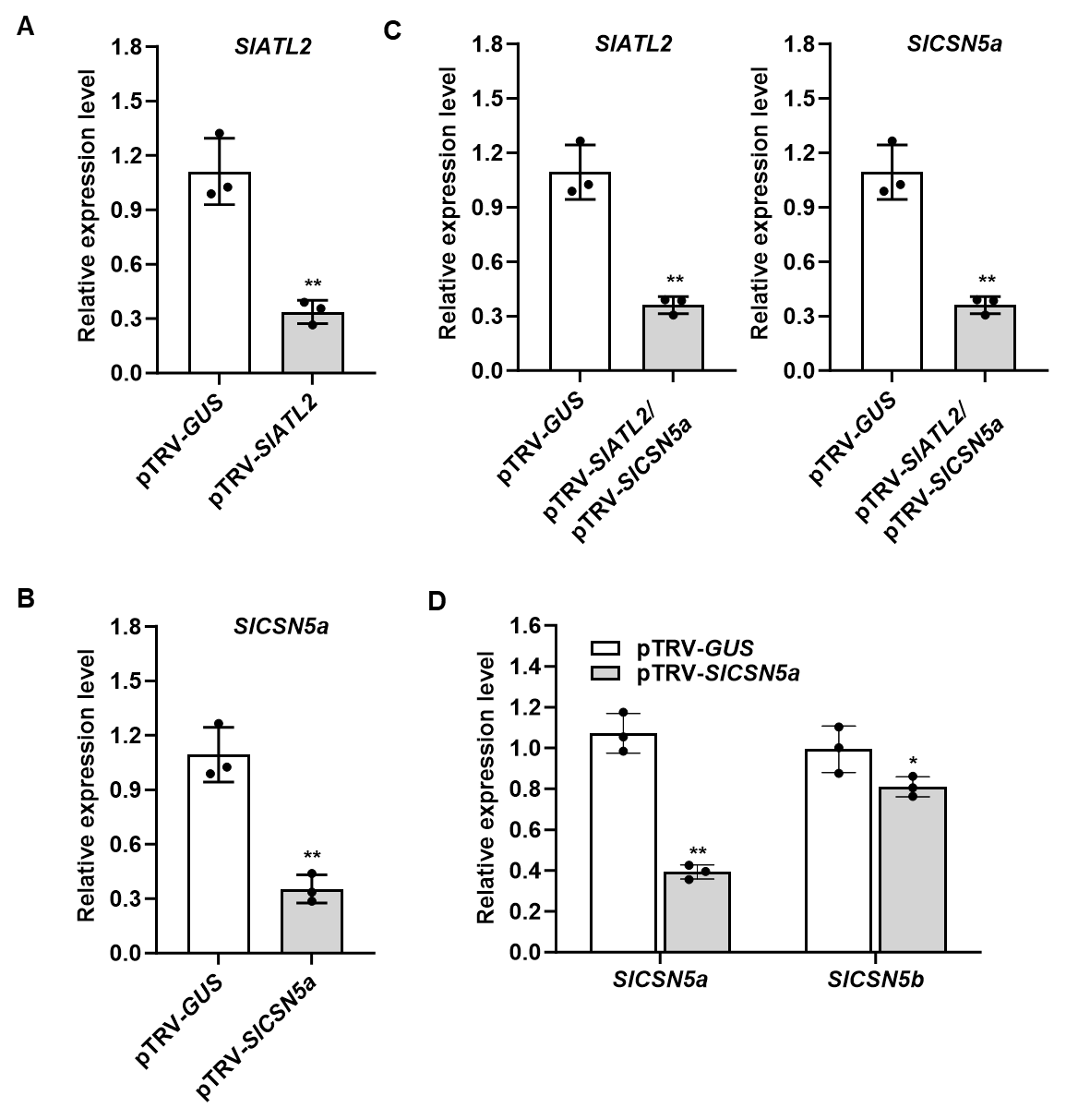


**Figure S5.** Silencing efficiency and specificity for target genes. (A) Silencing efficiency of *SlATL2* gene in pTRV-*SlATL2*-infiltrated plants. (B) Silencing efficiency of *SlCSN5a* gene in pTRV-*SlCSN5a*-infiltrated plants (C) Silencing efficiency of *SlATL2* or *SlCSN5a* gene in pTRV-*SlATL2*/pTRV-*SlCSN5a*-infiltrated plants. (D) Silencing specificity of *SlCSN5* genes in pTRV-*SlCSN5a*-infiltrated plants. Transcript levels of *SlATL2* and *SlCSN5a* genes in corresponding pTRV-*SlATL2*- or pTRV-*SlCSN5a*- or pTRV-*SlATL2*/pTRV-*SlCSN5a*-infiltrated and pTRV-*GUS*-infiltrated plants were analyzed by qRT-PCR. Data were shown as means ± SD; n =3. Asterisks represented statistical significance (**P* < 0.05, ***P* < 0.01, Student’s *t*-test). Experiments were repeated three times with similar results.


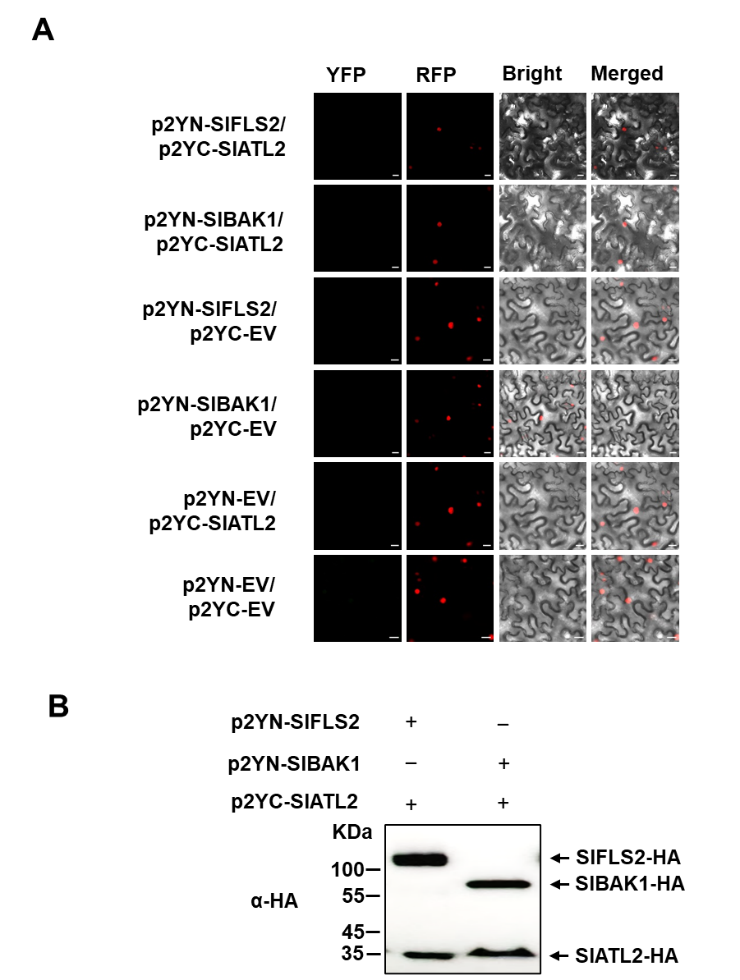


**Figure S6.** BiFC analysis of SlATL2 interaction with SlFLS2 and SlBAK1. (A)Various combinations of constructs were co-agroinfiltrated into leaves of *N. benthamiana* and the fluorescence signal was observed 2 days after agroinfiltration. Bars = 50 µm. (B) Accumulation of SlATL2, SlFLS2 and SlBAK1 in *N. benthamiana* leaves in BiFC. Fusion proteins were determined by immunoblot analysis with anti-HA antibody. All experiments were conducted at least three times with similar results.

**Figure S7.** Phylogenetic tree analysis of SlATL2 with *Arabidopsis* and rice ATL proteins. Phylogenetic tree was constructed by neighbor-joining method using MEGA7 program. The gene locus of *Arabidopsis* and rice *ATL* genes are as follows: *Arabidopsis thaliana* AtATL1 (AT1G04360), AtATL2 (AT3G16720), AtATL5 (AT3G62690), AtATL6 (AT3G05200), AtATL8 (AT1G76410), AtATL9 (AT2G35000), AtATL12 (AT2G20030), AtATL15 (AT1G22500), AtATL31 (AT5G27420), AtATL38 (AT2G34990), AtATL43 (AT5G05810), AtATL54 (AT1G72220), AtALT55(AT5G10380), AtATL61 (AT3G14320), AtATL62 (AT3G19140), AtATL78 (AT1G49230), AtATL80 (AT1G20823), *Oryza sativa* OsEL5/OsATL24 (LOC_Os02g35329), OsATL53 ((LOC_Os04g48310), OsBIRF1 (LOC_ Os02g50930). Bootstrap values from 1000 replicates are indicated at each node. Bar represents the number of amino acid differences per site.


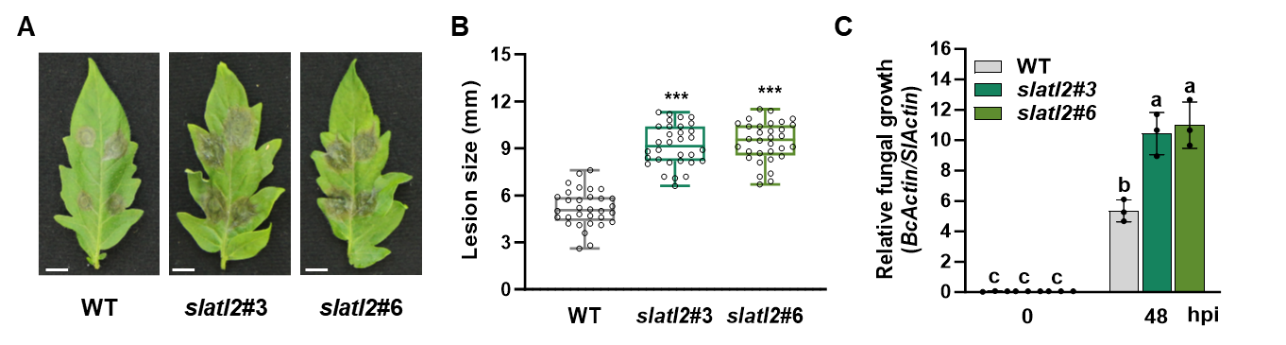


**Figure S8.** Knockout of *SlATL2* resulted in reduced resistance to *B. cinerea*. Four-week-old tomato plants were inoculated with a *B. cinerea* spore suspension (1 × 10⁵ spores/mL) by applying droplets onto detached leaves. Disease phenotype (A), lesion sizes (B) and fungal growth (C) in detached leaf inoculation assays. Photographs were taken at 3 days post-inoculation (dpi). Bars= 5 mm. Fungal growth *in planta* was indicated by the ratio of *BcActin* transcript level to that of *SlActin*. Data are shown as means ± SD; n = 32 (B) and n = 3 (C). Asterisks represented statistical significance (****P* < 0.001, Student’s *t*-test) (B). Statistically significant differences are denoted by different letters, as determined by two-way ANOVA followed by Tukey's test (*P*<0.05) (C). Experiments were repeated three times with similar results.


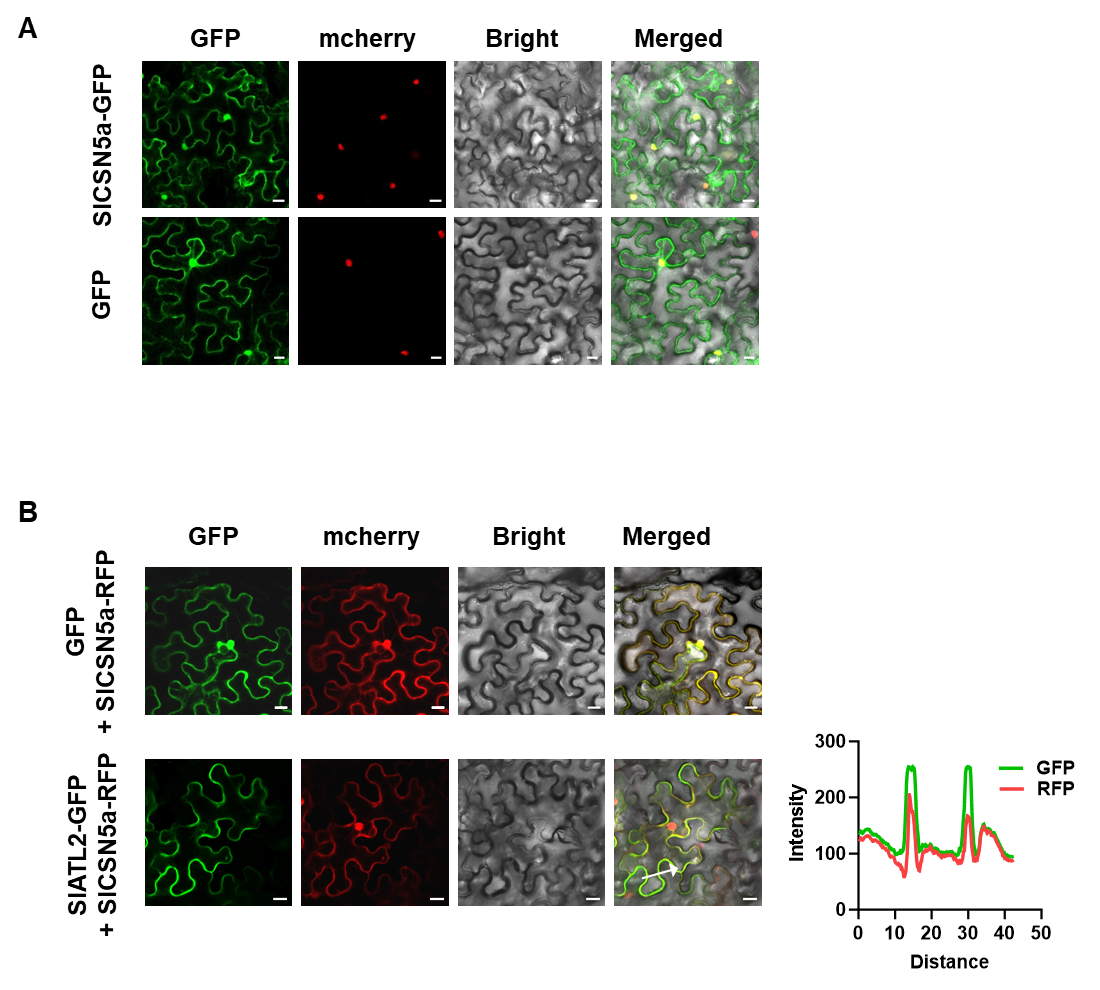


**Figure S9.** Subcellular localization and co-localization of SlATL2 and SlCSN5a. (A) Agrobacteria carrying pCAMBIA1300-SlCSN5a-GFP or pCAMBIA1300-GFP vector were infiltrated into leaves of *N. benthamiana* leaf epidermal cells expressing a red nucleus marker protein RFP-H2B. (B) *N. benthamiana* leaf epidermal cells were co-infiltrated with GFP or pCAMBIA1300-SlATL2-GFP and pCAMBIA1300-SlCSN5a-RFP. GFP or RFP fluorescence was examined under a confocal laser scanning microscope 48 hours later. Left, green fluorescence; middle left, red fluorescence; middle right, white field; right, merged images. Scale bars = 20 μm.

**Table S1 Putative SlATL2 interactors identified by Y2H screening.**

| **Proteins** | **Gene locus** | **No. of clones** |
| --- | --- | --- |
| **COP9 signalosome complex subunit CSN5a** | Solyc06g073150 | 22 |
| Protein-tyrosine-phosphatase PTP1 | Solyc11g016990 | 12 |
| PH-like superfamily protein | Solyc02g005603 | 6 |
| Ethylene-responsive TF ERF098-like | Solyc09g066350 | 5 |
| C1 domain | Solyc01g073800 | 4 |
| Myb-like DNA-binding domain | Solyc09g008250 | 4 |
| CoA binding domain | Solyc01g007910 | 3 |
| Ribosomal protein L35 | Solyc06g066190 | 3 |
| VQ motif family protein | Solyc06g060470 | 2 |
| **TOTAL** |  | **61** |

**Table S2 Sequence of the VIGS fragments for *SlATL2* and *SlCSN5a* genes**

| **Genes** | **Sequences for VIGS fragments (5’-3’)** | **Size (bp)** |
| --- | --- | --- |
| ***SlATL2*** | ATGAATAACGATACCCCAAGCATGATTCATGATCCATTGATTGATTCCACAGGAGATGGAGGCTATGCATTGAGTGGAAAAATTATGCTAAGTGCTATTGTTATCTTATTCACTGTTGTTGTTTTCATGGTGGGTCTTCATTTATATGCTCGTTGGTACCTTGTGTCTCAGCGCCGCCGTGAACTCCTTCGCCGCCGTAGGGTCAACCATCGACGGACTCATATTGTTTTCTACGTTGATAATCCTGGTTCCGGTTTGTCTGATGCTAACCGGGGTTTGGAACAGGCGGTTCTCAGTTCCTTGCCGGTTTTTGTTTATTCCGACAAGACTCACCCGGAACCGCTGGAATG | 350 |
| ***SlCSN5a*** | GATTCGAATTCAAAAAGATATTTAGAGTTCTTTTCTTAATTCAAGTAAATTTGTTCTAATTAACCCTGGTCGATTTTTAACTTATAAATTCAGATTGTAGCGCAGTGCTCGGCAATTGCCCTTCTCTCTGAAGTTACAATGGACTCTCTGAATTCTTACGCATCGTCGGCGGCGATGGCGCAGCAAACCTGGGAGTTAGAGAACAACATCGTAACAATCGACGCGCCGTCGGGGTCGAAACCGGAAAACTCCGCGTCGGACGCTATATTCCACTACGACGATGCGGCACAGACGAAGTTTCAG | 304 |

**Table S3 Primers used in this study for different purposes.**

| **Primers** | **Sequences (5’-3’)** |
| --- | --- |
| ***Subcellular Localization*** | |
| SlATL2-GFP-F | agctttcgcgagctcggtaccATGAATAACGATACCCCAAGCATG |
| SlATL2-GFP-R | gcccttgctcaccatggtaccCGGAGTTGTGATGCGTTCG |
| SlCSN5a-GFP-F | agctttcgcgagctcggtaccATGGACTCTCTGAATTCTTACGCA |
| SlCSN5a-GFP-R | gcccttgctcaccatggtaccGCTTTCGATCATGGGCTCG |
| SlCSN5a-RFP-F | agctttcgcgagctcggtaccATGGACTCTCTGAATTCTTACGCA |
| SlCSN5a-RFP-R | gcccttgctcaccatggtaccGCTTTCGATCATGGGCTCG |
| AtFLS2-GFP-F | agctttcgcgagctcggtaccATGAAGTTACTCTCAAAGACCTTTTTGA |
| AtFLS2-GFP-R | gcccttgctcaccatggtaccAACTTCTCGATCCTCGTTACGATC |
| ***VIGS constructs*** | |
| SlATL2-VIGS-F | CCCTCTAGAATGAATAACGATACCCCAAGCA |
| SlATL2-VIGS-R | GGGCTCGAGGGTTCCGGGTGAGTCTTGTC |
| SlCSN5a-VIGS-F | CCCTCTAGAGATTCGAATTCAAAAAGATATTTA |
| SlCSN5a-VIGS-R | GGGCTCGAGCTGAAACTTCGTCTGTGCC |
| ***Generation of transgenic tomato /Co-Immunoprecipitation*** | |
| SlATL2-OE-HA-F | ttacaattaccatggggcgcgccATGAATAACGATACCCCAAGCATG |
| SlATL2-OE-HA-R | aacatcgtatgggtaggtaccCGGAGTTGTGATGCGTTCG |
| SlATL2-CRISPR-F | CTGGTCTCTATTGaacaaagcaccagtggtctagtg |
| SlATL2-CRISPR-R | CTGGTCTCTCCGTAGGGTCAACCATCGACtgcaccagccgggaa |
| ***Yeast transformation*** | |
| SlATL2-AD-F | gccatggaggccagtgaattcATGAATAACGATACCCCAAGCATG |
| SlATL2-AD-R | cagctcgagctcgatggatccTCACGGAGTTGTGATGCGTTC |
| SlATL2-ΔTM-AD-F | gccatggaggccagtgaattcCGTTGGTACCTTGTGTCTCAGCG |
| SlATL2-ΔTM-AD-R | cagctcgagctcgatggatccTCACGGAGTTGTGATGCGTTC |
| SlCSN5a-BD-F | atggccatggaggccgaattcATGGACTCTCTGAATTCTTACGCA |
| SlCSN5a-BD-R | ccgctgcaggtcgacggatccTCAGCTTTCGATCATGGGCT |
| SlCSN5b-BD-F | atggccatggaggccgaattcATGGACGCTCTGAATTCTTACGC |
| SlCSN5b-BD-R | ccgctgcaggtcgacggatccTCAGGTTTCGACCATCGGC |
| SlATL2-N-AD-F | gccatggaggccagtgaattcATGAATAACGATACCCCAAGCATG |
| SlATL2-N-AD-R | cagctcgagctcgatggatccTTCCAGCGGTTCCGGGTG |
| SlATL2-RING-AD-F | gccatggaggccagtgaattcTGTGCTGTTTGTTTATCGGAATTT |
| SlATL2-RING-AD-R | cagctcgagctcgatggatccACAAAGAGGGCACGTAGAATGAG |
| SlATL2-C-AD-F | gccatggaggccagtgaattcCGCTCTCCGGTGGAGGCA |
| SlATL2-C-AD-R | cagctcgagctcgatggatccTCACGGAGTTGTGATGCGTTC |
| SlATL2-N19-AD-F | gccatggaggccagtgaattcATGAATAACGATACCCCAAGCATG |
| SlATL2-N19-AD-R | cagctcgagctcgatggatccATCTCCTGTGGAATCAATCAATGG |
| ***BiFC and SLC*** | |
| SlATL2-p2YC-F | atttacgaacgatagttaattaacATGAATAACGATACCCCAAGCATG |
| SlATL2-p2YC-R | actgccacctcctccactagtCGGAGTTGTGATGCGTTCG |
| SlCSN5a-p2YN-F | atttacgaacgatagttaattaacATGGACTCTCTGAATTCTTACGCA |
| SlCSN5a-p2YN-R | actgccacctcctccactagtGCTTTCGATCATGGGCTCG |
| SlFLS2-p2YN-F | atttacgaacgatagttaattaacATGATGATGTTAAAGACAGTTGTATATGC |
| SlFLS2-p2YN-R | actgccacctcctcc actagtATCTTTTACCAAATGAGAAGGCATAC |
| SlBAK1-p2YN-F | atttacgaacgatagttaattaacATGGATCAGTCGGTGTTGGC |
| SlBAK1-p2YN-R | actgccacctcctcc actagtTCTTGGCCCTGACAACTCATCC |
| SlATL2-nLuc-F | acgggggacgagctcggtaccATGAATAACGATACCCCAAGCATG |
| SlATL2-nLuc-R | CGCGTACGAGATCTGGTCGACAGCGTAATCTGGAACGTCATATGG |
| SlCSN5a-cLuc-F | tacgcgtcccggggcggtaccATGGACTCTCTGAATTCTTACGCA |
| SlCSN5a-cLuc-R | ACGAAAGCTCTGCAGGTCGACTCAAGCGTAATCTGGAACGTCA |
| SlCSN5b-cLuc-F | tacgcgtcccggggcggtaccATGGACGCTCTGAATTCTTACGC |
| SlCSN5b-cLuc-R | ACGAAAGCTCTGCAGGTCGACTCAAGCGTAATCTGGAACGTCA |
| ***Procaryotic protein expression*** | |
| SlATL2-C136H138A-F | CCAAGgctaatgcCTCTTTTCATTCGGAATGTATTGATAT |
| SlATL2-C136H138A-F | AAGAGgcattagcCTTGGGTAAAACCCGACCCT |
| GST-SlATL2-F | gatctggttccgcgtggatccATGAATAACGATACCCCAAGCATG |
| GST-SlATL2-R | gatgcggccgctcgagtcgacTCACGGAGTTGTGATGCGTTC |
| GST-SlATL2^C136H138A^-F | gatctggttccgcgtggatccATGAATAACGATACCCCAAGCATG |
| GST-SlATL2^C136H138A^-R | gatgcggccgctcgagtcgacTCACGGAGTTGTGATGCGTTC |
| GST-SlCSN5a-F | gatctggttccgcgtggatccATGGACTCTCTGAATTCTTACGCA |
| GST-SlCSN5a-R | gatgcggccgctcgagtcgacTCAGCTTTCGATCATGGGCT |
| His-SlATL2-F | gccatggctgatatcggatccATGAATAACGATACCCCAAGCATG |
| His-SlATL2-R | gtggtggtggtggtgctcgagCGGAGTTGTGATGCGTTCG |
| His-SlCSN5a-F | gccatggctgatatcggatccATGGACTCTCTGAATTCTTACGCA |
| His-SlCSN5a-R | gtggtggtggtggtgctcgagTCAGCTTTCGATCATGGGCT |
| ***Ubiquitination and degradation Assay*** | |
| SlATL2^C136H138A^-HA-F | ttacaattaccatggggcgcgccATGAATAACGATACCCCAAGCATG |
| SlATL2^C136H138A^-HA-R | aacatcgtatgggtaggtaccCGGAGTTGTGATGCGTTCG |
| SlCSN5a-HA-F | TTACAATTACCATGGGGCGCGCCATGGACTCTCTGAATTCTTACGCA |
| SlCSN5a-HA-R | AACATCGTATGGGTAGGTACCGCTTTCGATCATGGGCTCG |
| ***qRT-PCR*** | |
| SlATL2-RT-F | GTATGAACAGAAAAACACCCACCG |
| SlATL2-RT-R | TTCCACCACCACTACTACCGCC |
| SlCSN5a-RT-1F | GTCATGGGACTCATGCAGGGTA |
| SlCSN5a-RT-1F | TTCAACGGGGAGGGCAAAAG |
| SlCSN5b-RT-1F | AAAACAACATCGTGACGACGGA |
| SlCSN5b-RT-1F | GCCGCATCGTCATAGTGGAATA |
| SlRboh1-RT-F | GTTGCTGCAGCCATTGTCAC |
| SlRboh1-RT-R | GGCTTGGGCCAAAATCATTC |
| SlCAT1-RT-F | CCCAGTTAATGCTCCCAAGTGT |
| SlCAT1-RT-R | AGGACGACAAGGATCAAACCTC |
| SlSOD1-RT-F | GGCCAATCTTTGACCCTTTATG |
| SlSOD1-RT-R | AAGTCCAGGAGCAAGTCCAGTT |
| SlGR1-RT-F | GATGATGAAATGCGAGCTGTAG |
| SlGR1-RT-R | TTTGTGTTAGGGAGACGACCAG |
| SlPR1a-RT-F | GGCAGGAACACCAAAGAAACCA |
| SlPR1a-RT-R | TGGCCTCTGGTCAGGTTTAAAG |
| SlPR1b-RT-F | CCGTGCAATTGTGGGTGTC |
| SlPR1b-RT-R | GAGTTGCGCCAGACTACTTG |
| SlNPR1-RT-F | TGTGGGAAAGATAGCAGCACG |
| SlNPR1-RT-R | GTCCACACAAACACACACATC |
| SlICS1-RT-F | TCGCCGGCATTCATTGGAAACA |
| SlICS1-RT-R | GCACTCCCGTACTATAGCAAAC |
| SlJAZ1-RT-F | GATTTTCCGGCTGATAAAGCTAA |
| SlJAZ1-RT-R | TCCGAAACTCGGAACCACCAA |
| SlPI I-RT-F | GTTGTACAAATGCCTGTGGTGAC |
| SlPI I-RT-R | GGTAAGAGTACATGAAGAGATGC |
| SlMYC2-RT-F | TAGCCACACTGGAGGCAAGATT |
| SlMYC2-RT-R | CTAGGTCTAATTCCATGAGCGC |
| SlPTI5-RT-F | ATTCGCGATTCGGCTAGACATGGT |
| SlPTI5-RT-R | AGTAGTGCCTTAGCACCTCGCATT |
| SlLrr22-RT-F | AAGATTGGAGGTTGCCATTGGAGC |
| SlLrr22-RT-R | ATCGCGATGAATGATCGGTGGAGT |
| SlActin-RT-F | CCAGGTATTGCTGATAGAATGAG |
| SlActin-RT-R | GAGCCTCCAATCCAGACAC |
